# Genome-wide investigation of the LARP gene family: focus on functional identification and transcriptome profiling of *ZmLARP6c1* in maize pollen

**DOI:** 10.1186/s12870-024-05054-z

**Published:** 2024-04-29

**Authors:** Xiaoqin Xiang, Qianxia Deng, Yi Zheng, Yi He, Dongpu Ji, Zuzana Vejlupkova, John E. Fowler, Lian Zhou

**Affiliations:** 1 College of Agronomy and Biotechnology, Maize Research Institute, Southwest University, Beibei, Chongqing, 400715 China; 2 Department of Botany and Plant Pathology, Oregon State University, Corvallis, OR 97331 USA; 3 Engineering Research Center of South Upland Agriculture, Ministry of Education, Chongqing, 400715 China

**Keywords:** LARP, Genome-wide investigation, Maize, Pollen germination, Transcriptome profiling

## Abstract

**Background:**

The La-related proteins (LARPs) are a superfamily of RNA-binding proteins associated with regulation of gene expression. Evidence points to an important role for post-transcriptional control of gene expression in germinating pollen tubes, which could be aided by RNA-binding proteins.

**Results:**

In this study, a genome-wide investigation of the LARP proteins in eight plant species was performed. The LARP proteins were classified into three families based on a phylogenetic analysis. The gene structure, conserved motifs, *cis*-acting elements in the promoter, and gene expression profiles were investigated to provide a comprehensive overview of the evolutionary history and potential functions of *ZmLARP* genes in maize. Moreover, *ZmLARP6c1* was specifically expressed in pollen and ZmLARP6c1 was localized to the nucleus and cytoplasm in maize protoplasts. Overexpression of *ZmLARP6c1* enhanced the percentage pollen germination compared with that of wild-type pollen. In addition, transcriptome profiling analysis revealed that differentially expressed genes included *PABP* homologous genes and genes involved in jasmonic acid and abscisic acid biosynthesis, metabolism, signaling pathways and response in a *Zmlarp6c1::Ds* mutant and *ZmLARP6c1-*overexpression line compared with the corresponding wild type.

**Conclusions:**

The findings provide a basis for further evolutionary and functional analyses, and provide insight into the critical regulatory function of *ZmLARP6c1* in maize pollen germination.

**Supplementary Information:**

The online version contains supplementary material available at 10.1186/s12870-024-05054-z.

## Background

RNA-binding proteins (RBPs) play critical roles in post-transcriptional regulation, including pre-mRNA processing, and mRNA stability, translation, longevity, transport, and localization [1]. In higher plants, RBPs, as mRNA regulators, are essential for gene expression during floral development and response to environmental stimuli [2–4]. Many RBPs harbor the RNA recognition motif (RRM), which is the most frequently represented RNA-binding domain (RBD) in plants. The La Motif (LAM) is another classic RBD, which is present in La proteins and La-related proteins (LARPs). Genuine La proteins were first identified in humans [5]. The LARPs form a large superfamily and are classified into five families, namely, La (genuine La; LARP3), LARP1, LARP4, LARP6, and LARP7, based on structural features and evolutionary history [6–8]. The highly conserved LAM is present in all members of the LARP superfamily, whereas the RRM type is family specific [6, 9, 10].

The La proteins contain three structured domains, comprising the LAM, RRM1 (a canonical RNA recognition motif), and RRM2 (an atypical RNA recognition motif) [11, 12]. Numerous studies have demonstrated that La proteins recognize the 3′-UUU-OH motif of RNA precursors and this is directly involved in La nuclear functions [7, 11–14]. In plants, the *AtLa1* gene participates in the maturation of tRNA and is required for completion of embryogenesis in *Arabidopsis* [15, 16]. The LARP1 proteins carry the LAM, RRM-5L, and DM15 (smart00684) domains [6]. In *Arabidopsis*, LARP1 is involved in leaf senescence and heat-induced mRNA decay [17, 18], and plays a role in translation [19]. To date, LARP7 and LARP4 proteins have been identified and function only in vertebrates. The LARP7 proteins most closely resemble genuine La proteins that target RNA polymerase III to promote RNA maturation [20–23]. The LARP4 proteins contain a poly(A)-binding protein (PABP)-interacting motif 2 (PAM2), which is associated with a reorganization of the LAM and therefore alters the RNA-binding properties [24]. In animal cells, LARP1 and LARP4 proteins bind to the 5′ and 3′ untranslated regions (UTRs) of mRNAs to regulate translation and degradation [25–27].

The LARP6 proteins carry a La-module, comprising a conserved LAM and a specific RRM, designated RRM-L3a in plants and RRM-L3b in vertebrates. A short-conserved motif, termed the LAM and S1 associated (LSA) motif, located at the C-terminus [6] is involved in mediating protein–protein interactions [28–31]. In mammals, LARP6 coordinates the translation of collagen subunits by binding to a 50 stem-loop in the 5′-UTR of type I collagen mRNAs in the endoplasmic reticulum [32–34]. In vascular plants, LARP6 proteins are of three evolutionary types, designated 6a, 6b, and 6c, and are encoded by three to six genes [26]. The *Arabidopsis thaliana* genome contains three *AtLARP6* genes: *AtLARP6a*, *AtLARP6b*, and *AtLARP6c*. Maize (*Zea mays* L.) has six genes encoding LARP6 family proteins, namely, *ZmLARP6a*, *ZmLARP6b1–b3*, and *ZmLARP6c1–c2*. Notably, certain plant LARP6 proteins, such as LARP6b and LARP6c, have acquired a PAM2 motif, which directly interacts with the major plant PABP, and also show differential RNA-binding activity in vitro [26]. In *Arabidopsis*, *AtLARP6c* regulates mRNA post-transcription during guidance of the pollen tube to the embryo sac in the ovule [35]. In maize, ZmLARP6c1 has an important male-specific function in the haploid gametophyte during the highly competitive phase of pollen tube germination and growth following pollination [36]. These two studies suggest that LARP6c has conserved molecular and cellular roles in maize and *Arabidopsis*.

The male gametophyte of flowering plants, the pollen grain, consists of two sperm cells and a vegetative cell, and regulation of gene expression is required for their functions. In maize, after pollen deposition on the silk, the pollen grain germinates a pollen tube, which grows towards to the embryo sac to deliver the sperm cells for double fertilization [37–39]. Numerous genes are specifically expressed in the male gametophyte, as revealed by analysis of the male gametophyte transcriptome in plants [40–44]. In higher plants, pollen germination and pollen tube elongation require changes in the pattern of gene expression that are partially dependent on post-transcriptional mechanisms [2–4, 45]. RNA-binding proteins are associated with post-transcriptional regulation [1, 46]. However, the mechanism of post-transcriptional regulation of male gametophyte gene expression to ensure proper pollen germination and pollen tube elongation in maize remains unclear.

In this study, we performed a comprehensive analysis of the LARP family in maize, including analysis of phylogenetic relationships, gene structure, motif composition, and *cis*-acting elements. We investigated the expression profiles of LARP genes in various maize tissues, particularly in the floral organs. One of these genes, *ZmLARP6c1*, which was specifically and highly expressed in pollen. The *Ds* transposable element insertional mutant of *ZmLARP6c1* was associated with reduced transmission when crossed as a male in our previous studies [36]. We analyzed the subcellular localization of ZmLARP6c1, generated transgenic lines overexpressing this protein, and determined pollen germination in vitro. Furthermore, we analyzed the transcriptome profile of the overexpression lines and the *Ds* mutant in comparison with the wild type. This study provides a foundation for further evolutionary and functional exploration, and provides valuable insight into the critical regulatory function of *ZmLARP6c1* in maize pollen germination.

## Materials and methods

### Identification of LARPs

An initial BLAST search was performed using the LAM of the maize LARP protein as the query sequence. The LARP protein sequences of eight plant species, comprising *Zea mays*, *Arabidopsis thaliana*, *Sorghum bicolor*, *Oryza sativa*, *Glycine max*, *Hordeum vulgare*, *Triticum aestivum*, and *Nicotiana tabacum*, were downloaded from the NCBI databases (https://www.ncbi.nlm.nih.gov/) and the UniProt database (https://www.uniprot.org/). The candidate genes were searched by BLASTP using a score value ≥ 100 and e-value ≤ e^−10^. The NCBI CD-Search Tool (https://www.ncbi.nlm.nih.gov/Structure/bwrpsb/bwrpsb.cgi) was used to predict and determine the protein sequences. The basic properties of the identified LARP proteins were analyzed using the Expasy ProtParam tool (http://web.expasy.org/protparam/).

## Phylogenetic and sequence analysis

A multiple sequence alignment of the LARP amino acid sequences from maize and the other plant species was generated using MegAlign software with the ClustalW method. The phylogenetic analysis was performed using the maximum likelihood (ML) method in MEGA (https://www.megasoftware.net/). Then the phylogenetic tree was edited and visualized. The exon–intron structure of the *ZmLARP* genes was determined using the Gene Structure Display Server (https://gsds.gao-lab.org/). The conserved motifs of the LARP proteins were evaluated with the MEME Suite (https://meme-suite.org/meme/tools/meme).

### *Cis*‑acting element prediction

The 2000 bp promoter sequence upstream of the CDS of each *ZmLARP* gene was extracted using the gtf/gff3 sequence extraction tool in the TBtools [47] software. The promoter sequences were submitted to the PlantCare database (http://bioinformatics.psb.ugent.be/webtools/plantcare/html/) for *cis*-acting element prediction. The *cis*-acting elements of the *ZmLARP* genes were visualized using the biological sequence viewing tool in TBtools.

### Vector construction

For the subcellular localization assays, the CDS of *ZmLARP6c1* was fused with *GFP* and ligated into the pAN580 vector. For overexpression vector construction, the full-length CDS of *ZmLARP6c1* was amplified using maize mature pollen cDNA as the template. The target fragment was fused with a Myc tag and ligated into the modified plant transformation vector pCAMBIA3301 under the control of the ubiquitin promoter.

### Subcellular localization

Maize seeds were germinated and seedlings were grown in a growth chamber (30°C in the dark) to the V1 stage. Maize protoplasts were isolated from seedlings and transformed using polyethylene glycol (PEG). The plasmids harboring the ZmLARP6c1-GFP construct and RFP visualization marker were co-transformed into the protoplast suspension and then incubated for 16 h. A confocal laser-scanning microscope (LSM800, Carl Zeiss, Germany) was used for detection of the fluorescence signals. Three independent experiments were performed, with a negative control used in each experiment.

### Plant transformation

The overexpression vector was transformed into *Agrobacterium tumefaciens* strain EHA105 and then transformed into the maize inbred line B104 using the maize immature embryo transformation system. Immature embryos collected from ears at 11–12 days after pollination were transfected and then transferred to the co-cultivation medium for incubation. The embryos were screened and regenerated on selection and regeneration medium (containing 5 μg L^−1^ glufosinate ammonium; Sigma, USA). The regenerated plantlets were transplanted into pots containing soil and grown in a greenhouse. The T_1_ and T_2_ transgenic plants were identified by PCR, qRT-PCR, and western blotting. The T_3_ homozygous transgenic plants were used for further experiments. All primers used in this study are listed in Supplementary Table S1. The *Zmlarp6c1::Ds* allele used in this study was obtained from the Maize Genetics Cooperation Stock Center as previously described [36].

### In vitro pollen germination and pollen diameter measurement

Maize materials were germinated and cultivated in a manure ball, then the seedlings were transplanted and grown at the SWU Farm in Chongqing, China. Plants used for pollen germination experiments were grown under summer field conditions at the SWU Farm. Fresh mature pollen was collected between 11:00 am and 12:00 pm. Pollen was germinated on PGM (0.0005% H_3_BO_3_, 10 mM CaCl_2_, 0.05 mM KH_2_PO_4_, 6% PEG 4000, and 10% sucrose), and observed at 15 or 30 min after plating on the medium as described previously [36]. Germinated, non-germinated, and ruptured pollen grains were counted at each time point. Pollen tube length was measured at 30 min after plating on the medium. Four replicates were measured, with four *Zmlarp6c1::Ds* and four WT plants, or four *ZmLARP6c1-*OE and four WT plants used per replicate. A minimum of 150 pollen grains were categorized at each time point, and a minimum of 100 pollen tubes were measured for each experiment.

For measurement of the pollen grain diameter, fresh pollen was collected from *ZmLARP6c1-*OE and WT plants, immediately fixed in ethanol:acetic (3:1) acid solution, and rehydrated for measurement of the pollen grain diameter as described previously [36]. Four replicates were measured, with at least 150 pollen grains measured per replicate.

### RNA extraction, real-time qRT-PCR, and RNA-Seq analysis

Maize plants used for RNA extraction were grown under summer field conditions at the SWU Farm. Fresh mature pollen was collected between 11:00 am and 12:00 pm and frozen in liquid nitrogen. Total RNA isolation, cDNAs synthesis, and real-time qRT-PCR were performed as described previously [36]. Primers used for real-time qRT-PCR are listed in Supplementary Table S1.

RNA-Seq was performed using three biological replicates for each genotype. Sequencing was conducted with the Illumina sequencing library of the Beijing BioMarker Technologies Corporation (Beijing, China). Differentially expressed genes between two comparative groups were identified with the DESeq2 software. The differentially expressed genes/transcripts were filtered based on a *P*-value < 0.01 after adjustment to control the FDR.

### Statistical analysis

All experiments were repeated at least three times and yielded similar results. Statistical significance was determined using Student’s *t*-test to compare the three pollen germination categories. A significant difference was determined at the 0.05, 0.01, or 0.001 significance levels.

## Results

### Phylogenetic analysis and classification of LARPs in eight plant species

**Fig. 1:**
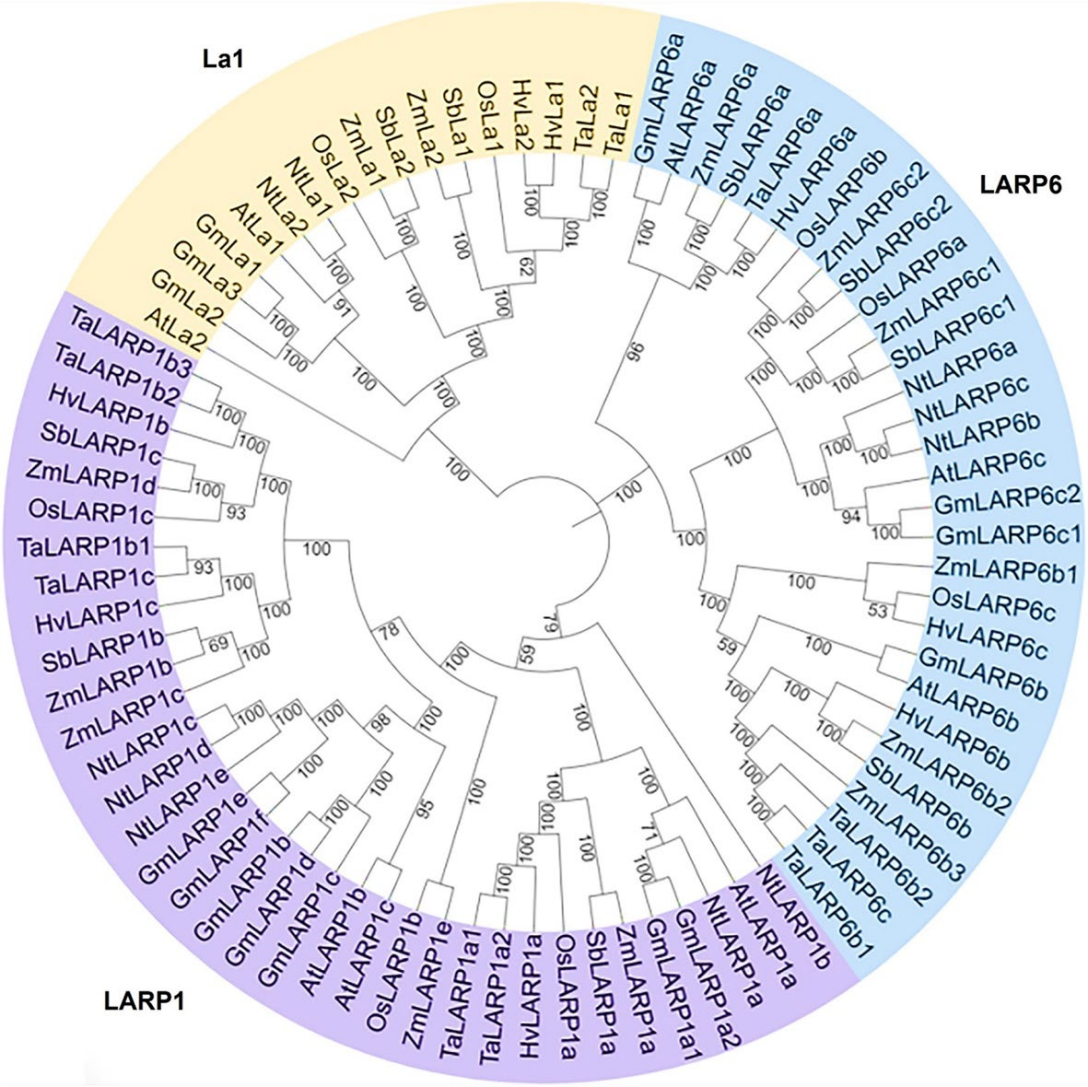
Phylogenetic tree and family classification of the LARP proteins from eight plant species. The LARP1, La, and LARP6 families are marked with different colors. Prefix “Zm” indicates “*Zea mays*”, “At” indicates “*Arabidopsis thaliana*”, “Gm” indicates “*Glycine max*”, “Hv” indicates “*Hordeum vulgare*”, “Nt” indicates “*Nicotiana tabacum*”, “Os” indicates “*Oryza sativa*”, “Sb” indicates “*Sorghum bicolor*” and “Ta” indicates “*Triticum aestivum*”

A total of 82 LARP proteins were identified in eight plant species, comprising 13 in *Zea mays*, 8 in *Arabidopsis thaliana*, 9 in *Sorghum bicolor*, 8 in *Oryza sativa*, 14 in *Glycine max*, 8 in *Hordeum vulgare*, 12 in *Triticum aestivum*, and 10 in *Nicotiana tabacum* (Table S2). To examine the phylogenetic relationships of the LARP proteins, we constructed a phylogenetic tree based on a multiple sequence alignment of the LARP proteins of maize and the other plant species. The phylogeny sorted the LARP proteins into groups corresponding to three families, namely, LARP1, La (genuine La; LARP3), and LARP6 (Fig. 1; Table S3). The LARP4 and LARP7 proteins were not present in these plant species (Fig. 1). In maize, the LARP6 family had the highest representation and was further categorized into three major groups, namely, LARP6a, 6b, and 6c.

### Gene structure and motif analysis of LARPs in maize

By comparing the genomic DNA sequences, we determined the exon and intron structure of the maize *LARP* genes. The coding sequences (CDSs) of all genes included introns, with the number of exons ranged from 6 to 14 (Fig. S1A). The number of exons was conserved in each *LARP* family: 6 exons in most *ZmLARP1* genes (*ZmLARP1b*–*ZmLARP1e*), except for 14 exons in *ZmLARP1a*; 10 exons in *ZmLa* genes; and 10 exons in most *ZmLARP6* genes, except for 9 exons in *ZmLARP6c1*. The exon length was similar within a *LARP* family, although the positions of the exons varied among individual genes. In general, members with close relationships from the same family shared similar exon numbers and exon lengths.

A multiple sequence alignment of LARP proteins was generated based on the LAM domain. Further analysis showed the LARP1 proteins harbored the LAM, among which one protein, ZmLARP1a, had a DM15-ABC domain (Figs. S1B and S2). The La proteins contained the LAM, RRM1, and RRM2 domains (Figs. S1B and S3). The LARP6 proteins contained the LAM, RRM-L3a, and LSA elements were highly conserved (Figs. S1B and S4). Notably, members of the LARP6b and LARP6c subgroups had acquired a PAM2 motif in the N-terminal region (Figs. S1B and S4). These conserved amino acid profiles may contribute to the classification of LARP genes in other plant species.

### Analysis of *cis*-acting elements in the *LARP* promoter in maize

To further study the regulatory mechanism of the *ZmLARP* genes during environmental adaptation, the 2 kb sequences upstream of the 13 *ZmLARP* genes with complete domains were extracted from the maize reference genome for *cis*-acting element analysis (Fig. S5A; Table S4). Approximately 43 *cis*-acting elements with unambiguous functions were analyzed. Light-responsive elements, comprising G-box, Sp1, GT1-motif, MRE, TCCC-motif, TCT-motif, GATA-motif, AE-box, AAAC-motif, ATCT-motif, ATC-motif, GA-motif, I-box, chs-CMA1a, LAMP-element, GTGGC-motif, Box II, Box 4, ACA-motif, TCT-motif, and ACE motifs, were predominant. Hormone-associated elements present included those responsive to methyl jasmonate (MeJA) (CGTCA-motif and TGACG-motif), abscisic acid (ABRE), gibberellin (GARE-motif, P-box, and TATC-box), salicylic acid (TCA-element), and auxin (TGA-element and AuxRR-core). Abiotic stress-response elements contained MBS (drought inducibility), LTR (low-temperature responsiveness), and TC-rich repeats (defense and stress responsiveness). Other response elements detected included the A-box (*cis*-acting regulatory element), ARE (anaerobic induction), MSA-like (cell-cycle regulation), AT-rich (binding site of AT-rich DNA-binding proteins), RY-element (seed-specific regulation), O2-site (zein metabolism regulation), CAT box (meristem expression), GCN4-motif (endosperm expression), GC-motif (anoxic specific inducibility), HD-Zip 1 (differentiation of the palisade mesophyll cells), and CCAAT-box (MYBHv1 binding site). Light-responsive, MeJA-responsive, and abscisic acid (ABA)-responsive elements were the most abundant *cis*-acting elements detected in the ZmLARP proteins (Fig. S5B). These data may hint at the diverse functions of ZmLARP proteins in maize.

### *LARP* gene expression profile in different tissues of maize

**Fig. 2:**
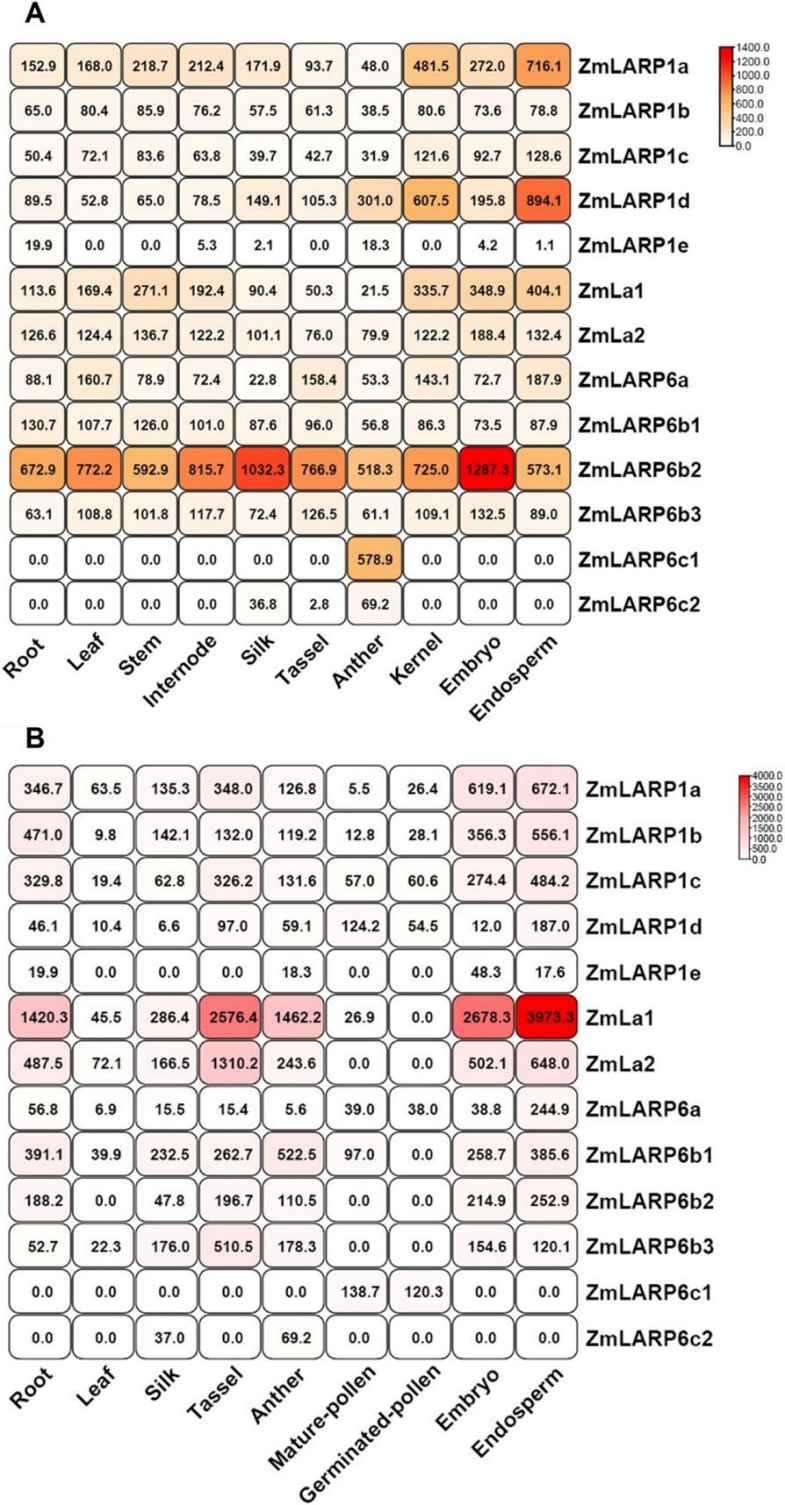
**A** Transcript level and **B** protein abundance of ZmLARPs in different tissues of maize. Germinated pollen and mature pollen samples were assessed for proteomic data. White-to-red cell color is representative of FPKM, with the value shown in the center of each cell

To evaluate the potential functions of *ZmLARP* genes in different tissues of maize, transcriptional (Fig. 2A) and proteomic (Fig. 2B) profiling data from the Walley [48] maize developmental atlas were used to examine their expression patterns. Most *ZmLARP* genes were expressed in almost all tissues analyzed, suggesting that *ZmLARP* genes function in diverse maize organs. Notably, at the transcriptional and proteomic levels, *ZmLARP6c1* had the highest abundance among the *ZmLARP* genes in the anther and pollen. Transcripts of *ZmLARP6c1* were detected only in the anther (which includes developing pollen grains), and the translated protein was detected only in pollen, suggesting that *ZmLARP6c1* is specifically enriched in pollen, consistent with its documented function in maize pollen growth and development [36]. Intriguingly, *ZmLARP6c2*, the closest paralog, is enriched not only in male reproductive organs (anther, pollen), but also in silk, a female reproductive structure, raising the possibility that the *ZmLARP6c* clade specifically supports reproductive functions in maize. The transcriptomic and proteomic abundance of *ZmLARP* genes varied in different tissues, suggesting that *ZmLARP* genes exhibit diverse functions during the growth and development of maize.

### Subcellular localization of ZmLARP6c1

**Fig. 3:**
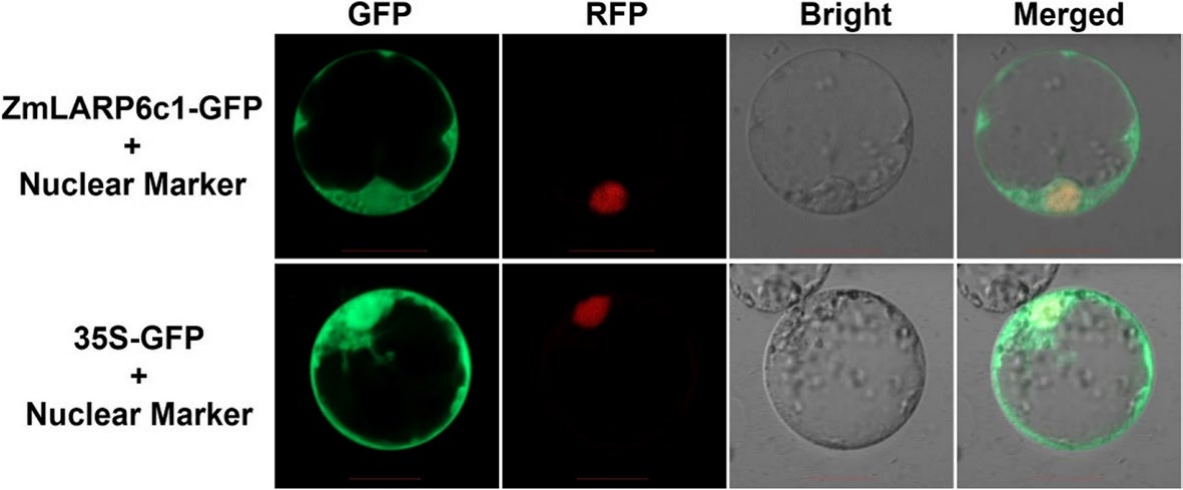
Subcellular localization of ZmLARP6c1. GFP, green fluorescence protein; RFP, red fluorescence protein. Scale bars = 20 μm. 35S-GFP served as the negative control

Our previous study revealed that *ZmLARP6c1* was specifically expressed in pollen, performed an important male-specific function in pollen tube germination and growth phase [36]. To investigate the subcellular localization of ZmLARP6c1, the full-length CDS of *ZmLARP6c1* was cloned and fused with *GFP* in the pAN580 vector, and transiently expressed in maize mesophyll protoplasts. The ZmLARP6c1-GFP fusion signal was detected in the nucleus and cytoplasm. To confirm nuclear localization, a nuclear-targeted RFP fusion protein was co-expressed with the GFP fusion. This pattern was similar to that of GFP alone (Fig. 3).

### Overexpression of *ZmLARP6c1* altered pollen germination

**Fig. 4:**
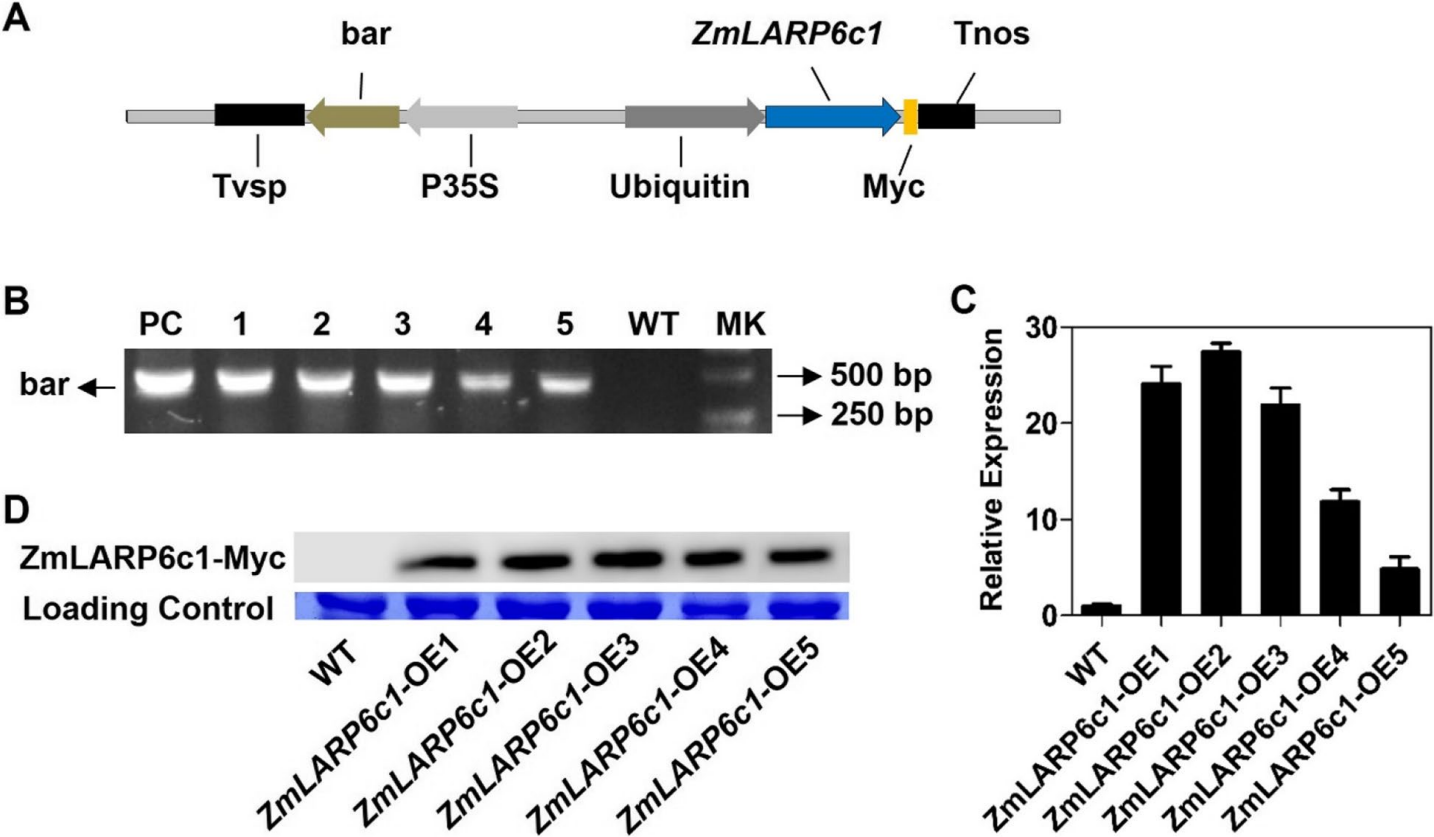
Characteristics of *ZmLARP6c1* overexpression lines of maize. **A** Schematic diagram of the *ZmLARP6c1*-OE vector. **B** Detection of *ZmLARP6c1*-OE transgenic lines by PCR. bar, bialaphos resistance gene; 1–5, *ZmLARP6c1*-OE1–OE5 lines; PC, positive control; MK, marker. **C** Expression analysis of *ZmLARP6c1*-OE lines by qRT-PCR. **D** Western blot analysis of *ZmLARP6c1*-OE lines

To further investigate the functional role of *ZmLARP6c1* in maize, independent transgenic lines that constitutively overexpressed *ZmLARP6c1* under the control of Ubiquitin promoter (Fig. 4A) were generated and identified by PCR (Fig. 4B), quantitative real-time PCR (qRT-PCR) (Fig. 4C), and western blotting, via the Myc-tag fused translationally to the ZmLARP6c1 protein C-terminus (Fig. 4D). Two representative overexpression lines were selected for further experiments (*ZmLARP6c1*-OE1 and *ZmLARP6c1*-OE2). The *ZmLARP6c1-*OE plants showed no obvious phenotypic difference from the wild type (WT) when grown in the field.

**Fig. 5:**
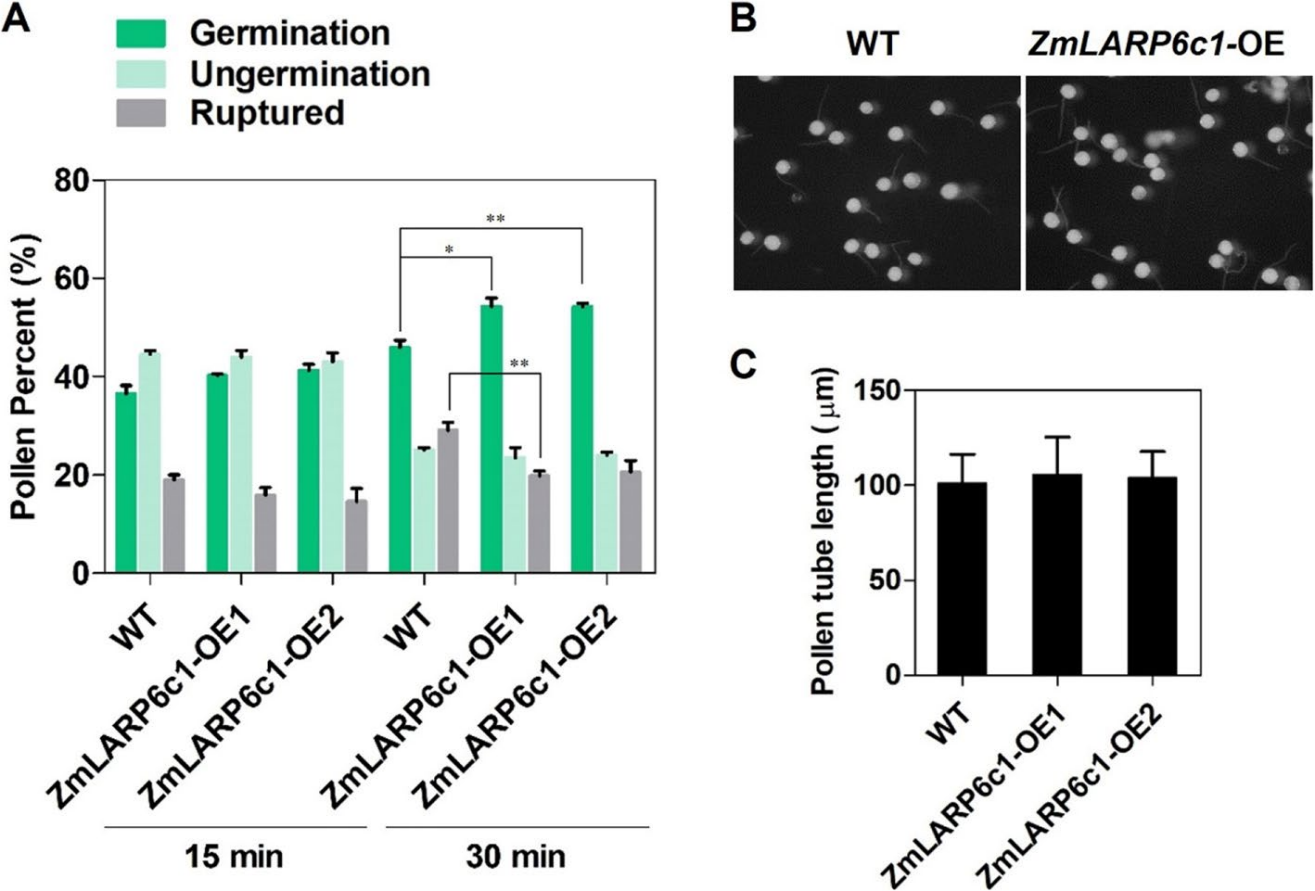
In vitro pollen germination and pollen tube growth. **A** Germinated, non-germinated, and ruptured pollen grains of *ZmLARP6c1*-OE and wild-type (WT) plants at 15 and 30 min after plating on pollen growth medium (PGM). Four biological replicates of four plants of each genotype, at least 150 pollen grains from each plant. **B** Representative fields-of-view of germinated pollen grains at 30 min after plating on PGM. **C** Pollen tube length of *ZmLARP6c1*-OE and the WT at 30 min after plating on PGM. A minimum of 100 pollen tubes were measured for each experiment. * *P* < 0.05, ** *P* < 0.01

We collected pollen samples from field-grown *ZmLARP6c1*-OE and WT (B104 inbred-line background) plants, characterized pollen germination and pollen tube growth in vitro (Fig. 5). The number of germinated, non-germinated, and ruptured pollen grains was scored at 15 and 30 min after plating on pollen growth medium (PGM). The percentage pollen germination was significantly higher in *ZmLARP6c1*-OE plants than in the WT (average 18.0% higher) at 30 min. The percentage of non-germinated pollen grains in *ZmLARP6c1*-OE plants and the WT did not differ significantly at 15 and 30 min, whereas the percentage of ruptured pollen grains was significantly lower in *ZmLARP6c1*-OE plants than in the WT at 30 min (Fig. 5A). Pollen tube lengths were observed and measured at 30 min after plating for all genotypes (Fig. 5B). No significant difference between *ZmLARP6c1*-OE plants and the WT was detected (Fig. 5C).

In the meanwhile, we characterized pollen germination and pollen tube growth of *Zmlarp6c1::Ds* (*tdsgR82C05*), a line carrying a *Ds-GFP* transposable element insertion in *ZmLARP6c1*, in SWU farm. The percentage pollen germination for *Zmlarp6c1::Ds* was significantly lower than that of the WT (W22 inbred-line background) (62.2% and 74.7%, respectively) at 15 and 30 min after plating. Conversely, the percentage of non-germinated pollen grains was higher in *Zmlarp6c1::Ds* than in the WT at 15 and 30 min (Fig. S6A). Pollen tube lengths were significantly shorter in *Zmlarp6c1::Ds* than in the WT at 30 min as previously described (Fig. S6B and C) [36].

No significant difference in pollen grain diameter was detected between the WT and *ZmLARP6c1-*OE pollen (Fig. S7). These assays indicated that overexpression of *ZmLARP6c1* influences the success of pollen germination, but not pollen tube growth, in maize. The shift from pollen grain rupture to successful pollen tube germination upon overexpression (Fig. 5A) raises the possibility that increases in *ZmLARP6c1* increase pollen resilience, at least in vitro.

### Analysis of transcriptome profiling in maize pollen

**Fig. 6:**
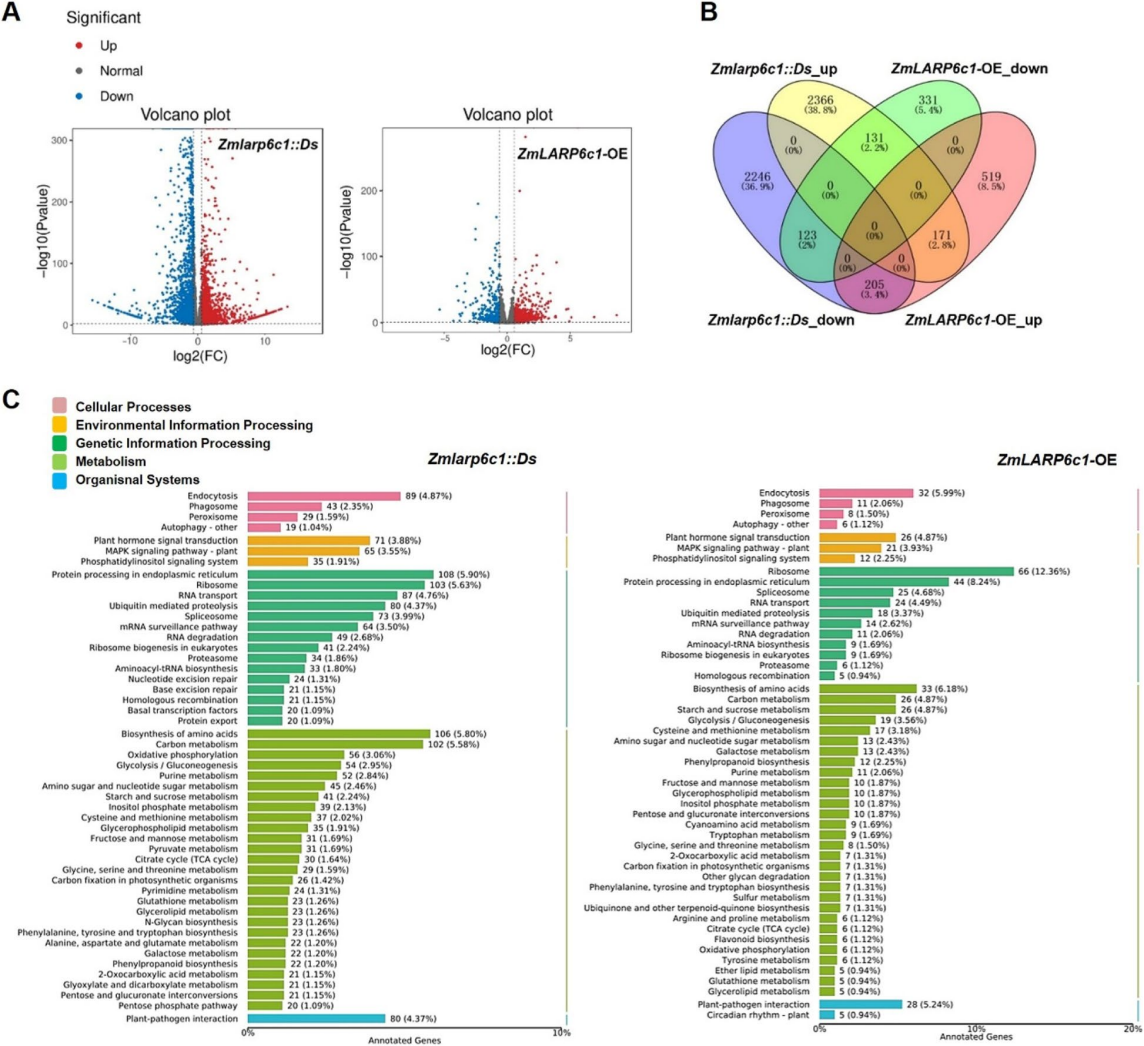
Differentially expressed genes (DEGs) in pollen of *Zmlarp6c1::Ds* and *ZmLARP6c1*-OE plants compared with the corresponding wild type (WT). **A** Volcano plot of DEGs. Red and blue dots represent up- and downregulated genes, respectively, in pollen of *Zmlarp6c1::Ds* and *ZmLARP6c1*-OE compared with WT. **B** Venn diagram showing the number of DEGs detected in pollen of *Zmlarp6c1::Ds* and *ZmLARP6c1*-OE. **C** KEGG pathway enrichment analysis of DEGs in pollen of *Zmlarp6c1::Ds* and *ZmLARP6c1*-OE compared with WT. Presented are the most significantly enriched pathways

To analyze the global transcriptional changes in the pollen caused altering the expression level of *ZmLARP6c1*, RNA-sequencing (RNA-Seq) analysis of the transcriptome of mature pollen from *Zmlarp6c1::Ds*, *ZmLARP6c1*-OE (*ZmLARP6c1-*OE1 and *ZmLARP6c1-*OE2), and the corresponding WT plants was conducted. In total, 5242 (2668 upregulated and 2574 downregulated) and 1480 (895 upregulated and 585 downregulated) differentially expressed genes (DEGs) were detected in *Zmlarp6c1::Ds* and *ZmLARP6c1*-OE, respectively, compared with the corresponding WT (Fig. 6A). The *Ds* insertion mutant and overexpression of *ZmLARP6c1* in the pollen strongly affected the number of DEGs. To further evaluate the function of *ZmLARP6c1* in the pollen, a crossover analysis of genes that were significantly up- or downregulated in *Zmlarp6c1::Ds* and *ZmLARP6c1*-OE compared with the WT plants was performed. We found 205 genes were upregulated in *ZmLARP6c1-*OE but downregulated in *Zmlarp6c1::Ds*, and 131 genes were downregulated in *ZmLARP6c1-*OE but upregulated in *Zmlarp6c1::Ds* (Fig. 6B). This is the expected pattern for genes whose expression is directly dependent on the level of *ZmLARP6c1* present in pollen. Somewhat surprisingly, a smaller, but still significant, number of genes were observed with similar expression outcomes in both *Zmlarp6c1::Ds* and *ZmLARP6c1*-OE – i.e., either upregulated (171 genes) or downregulated (123 genes) in both lines. This implies that *ZmLARP6c1* regulation of these genes is more complex than simple dose-dependence.

Gene Ontology (GO) functional annotation was implemented for DEGs in *Zmlarp6c1::Ds* and *ZmLARP6c1*-OE, and the significantly enriched GO terms (q value < 0.05) were shown in Table S5. The results suggested that these DEGs were involved in a wide range of processes. Notably, DEGs found in both genotypes were significantly enriched in several broadly-defined GO subcategories (Fig. S8). Intriguingly, given that La family proteins generally bind RNA to accomplish their regulatory functions, the GO term enrichment analysis revealed that 204 and 61 annotated DEGs (false discovery rate [FDR] < 0.01) were enriched in the subset of genes involved in RNA binding in *Zmlarp6c1::Ds* and *ZmLARP6c1*-OE, respectively, compared with the corresponding WT (Table S6). The ZmLARP6c1 protein carries a La-module, comprising a conserved LAM and a specific RRM-L3a domain, a short conserved LSA motif at the C-terminus, and a PAM2 motif at the N-terminus; on the basis of these motifs, differential RNA-binding activity is predicted. The data thus indicate that the level of *ZmLARP6c1* expression (either up or down) broadly affects the expression of other RNA-binding-related genes in the pollen. A further 72 and 20 annotated DEGs were enriched in the subset of genes categorized as associated with reproduction in *Zmlarp6c1::Ds* and *ZmLARP6c1*-OE, respectively, compared with the WT (Table S6). More specifically, 39 and 9 annotated DEGs were enriched in the subset of genes categorized as associated with pollen development in *Zmlarp6c1::Ds* and *ZmLARP6c1*-OE, respectively, compared with the WT (Table S7). Some of the enriched genes have been reported to impact plant growth and development. For example, the expression of a Tubby-like protein (TLP), Zm00001eb157040, was downregulated in *Zmlarp6c1::Ds* but was upregulated in *ZmLARP6c1*-OE. AtTLPs appear to function in multiple physiological and developmental processes in *Arabidopsis*: e.g., overexpression of *AtTLP2* enhances plant growth [49]; and AtTLP3 together with AtTLP9 modulates ABA- and osmotic stress-mediated seed germination [50]. Another example is the aquaporin ZmSIP2;1/sbip2a (Zm00001eb015230), which was downregulated in *Zmlarp6c1::Ds*. In *Arabidopsis*, a mutant of *Atsip2;1* was associated with defects in both pollen germination and pollen tube elongation, likely due to alleviation of ER stress by AtSIP2;1 [51].

**Fig. 7:**
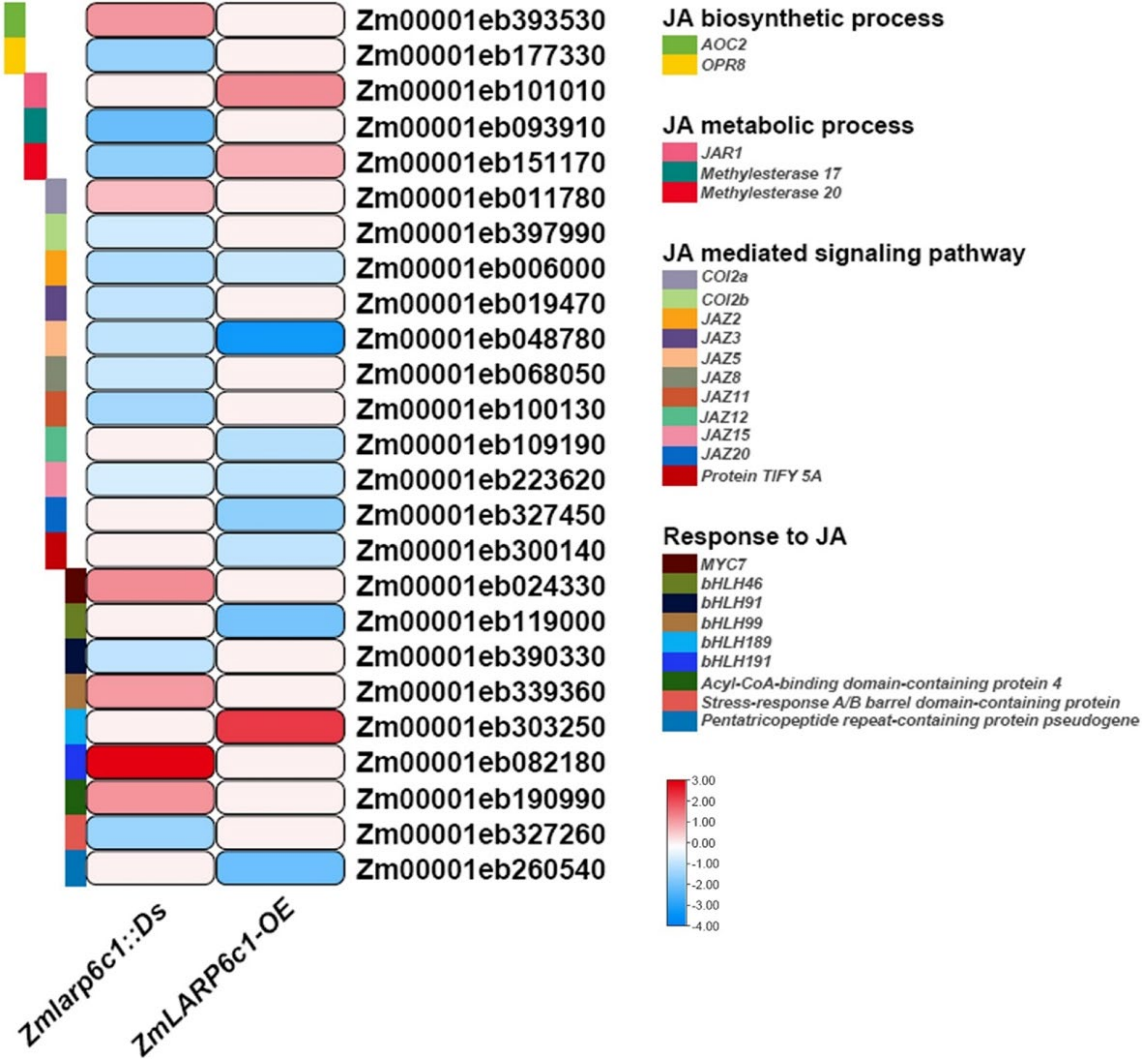
Log-fold change of differentially expressed genes associated with the jasmonic acid biosynthetic process, metabolic process, mediated signaling pathway, and response in pollen of *Zmlarp6c1::Ds* and *ZmLARP6c1*-OE compared with the wild type (WT). Columns represent comparison groups and rows represent genes. Low to high expression is indicated by color changes (blue to red)

**Fig. 8:**
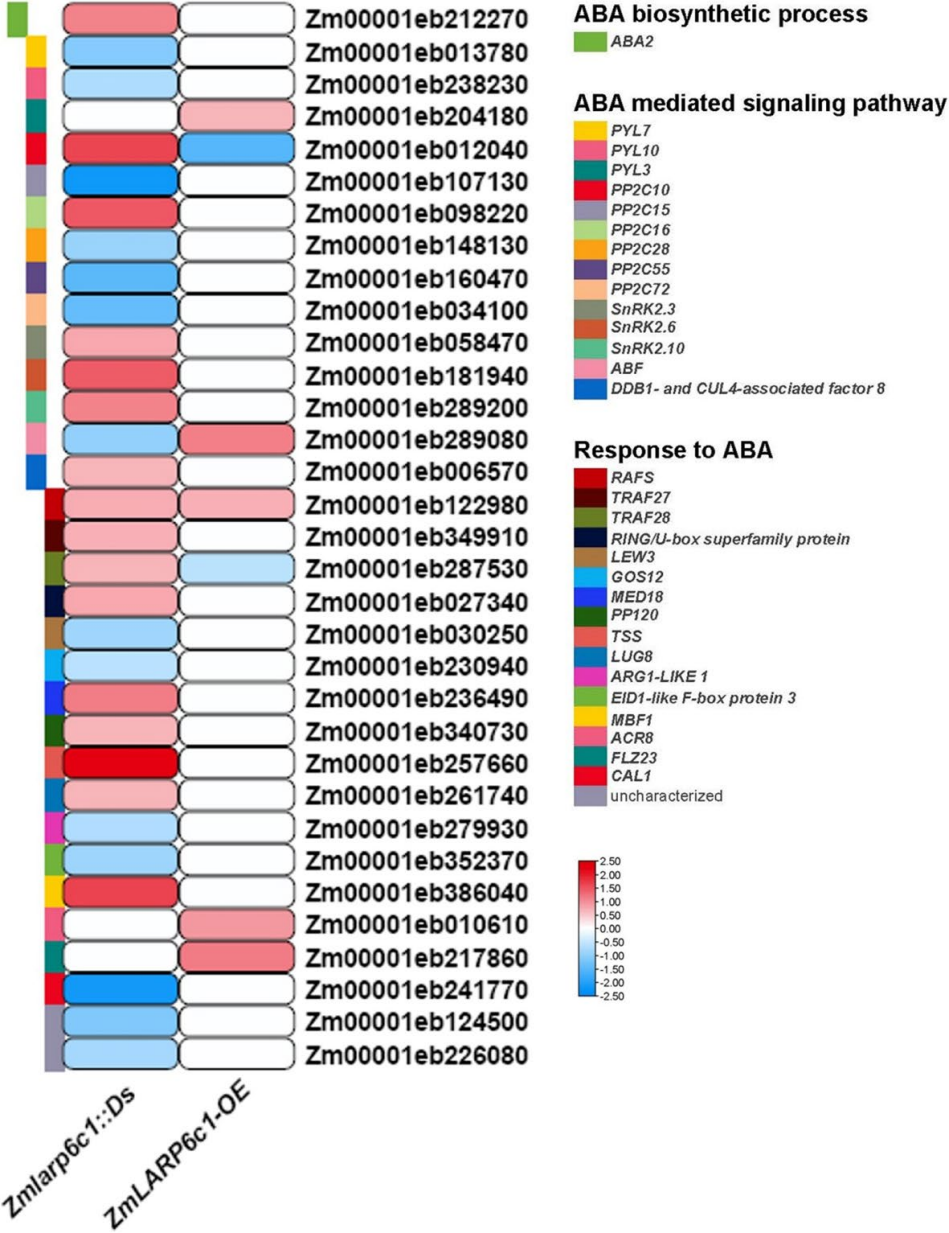
Log-fold change of differentially expressed genes associated with the abscisic acid biosynthetic process, mediated signaling pathway, and response in pollen of *Zmlarp6c1::Ds* and *ZmLARP6c1*-OE compared with the wild type (WT). Columns represent comparison groups and rows represent genes. Low to high expression is indicated by color changes (blue to red)

A KEGG pathway analysis of DEGs revealed enrichment of more specific downstream processes, potentially influenced by *ZmLARP6c1*: the ribosome, protein processing in the endoplasmic reticulum, RNA transport, spliceosome, ubiquitin-mediated proteolysis, and mRNA surveillance pathways in genetic information processing; the plant hormone signal transduction and MAPK signaling pathways in environmental information processing; and amino acid biosynthesis and carbon metabolism in metabolism (Fig. 6C). Within the genetic information processing category, the DEGs showed an association with the mRNA surveillance pathway: e.g., the expression levels of PABP homologous genes were up- and downregulated in *Zmlarp6c1::Ds* and *ZmLARP6c1*-OE, respectively (Table S8). Within the plant hormone signal transduction category, the DEGs were mainly associated with jasmonic acid (JA) and ABA biosynthesis, metabolism, signaling pathways, and response (Figs. 7 and 8; Table S8). In comparison with the WT, in the JA metabolic process, *allene oxide cyclase* (*AOC*) and *12-oxo-phytodienoic acid reductase* (*OPR*) homologous genes, Zm00001eb393530 and Zm00001eb177330, were up- and downregulated, respectively, in *Zmlarp6c1::Ds*. The JA biosynthetic process-related homologous gene, *jasmonic acid resistant 1* (*JAR1*; Zm00001eb101010), was upregulated in *ZmLARP6c1*-OE. Two homologous *methylesterase* genes were downregulated in *Zmlarp6c1::Ds*, but one of these genes was upregulated in *ZmLARP6c1*-OE. In the JA-mediated signaling pathway,* coronatine insensitive 1* (*COI1*) homologous genes (Zm00001eb011780 and Zm00001eb397990) were up- and downregulated in *Zmlarp6c1::Ds*, but were unchanged in *ZmLARP6c1*-OE. *Jasmonate ZIM domain* (*JAZ*) homologous genes were all downregulated in both *Zmlarp6c1::Ds* and *ZmLARP6c1*-OE. Among JA-responsive genes, *MYC* homologous genes were up- and downregulated in both *Zmlarp6c1::Ds* and *ZmLARP6c1*-OE (Fig. 7; Table S8). In the ABA signaling pathway, *PYL* homologous genes were downregulated in *Zmlarp6c1::Ds* (Zm00001eb013780, − 1.10; and Zm00001eb238230, − 0.73), and upregulated *ZmLARP6c1-*OE (Zm00001eb204180, 0.68). An ABRE binding factor (*ABF*) homologous gene (Zm00001eb289080) was downregulated in *Zmlarp6c1::Ds* (− 0.97) and upregulated *ZmLARP6c1*-OE (1.14) (Fig. 8; Table S8). These results suggested that *ZmLARP6c1* influences the JA and ABA biosynthetic and signaling systems in maize pollen. To corroborate the transcriptome profiling, we further analyzed the expression patterns of several representative JA and ABA biosynthetic, metabolic, signaling pathway, and responsive genes in mutant or overexpression plants and the corresponding WT by qRT-PCR. The results were consistent with the RNA-seq results (Fig. S9).

## Discussion

Not all LARP families are represented in all eukaryotes; however, the LAM is present in almost all families that are represented. In the present study, 82 LARP proteins were identified from eight plant species and assigned to three families: LARP1, La, and LARP6. The LARP4 and LARP7 families have not been reported in plants to date. The LARP1 proteins were the most numerous, and at least one member of the LARP1 family contained DM15 boxes in all eight plant species, whereas the other members had only one LAM (Table S3). The La proteins were the least numerous but had the most conserved domains, including LAM, RRM1, and RRM2, in all eight plant species. All LARP6 proteins carry a La-module, composed of the LAM and RRM-L3a domains, which is highly versatile and confers RNA-binding capacity [10, 52]. The LSA domain is located at the C-terminus of LARP6 (Table S3), which indicates a role in mediating protein–protein interactions [28–31]. The LARP6 proteins have acquired RG repeats, located between the LSA and RRM-L3a domains, which are predicted to be a nucleotide-binding motif [53]. The LARP6 proteins were further subdivided into three distinct subgroups that were designated 6a, 6b, and 6c. In this study, each plant species had a single LARP6a protein, and one to three 6b and 6c proteins (Table S3). Notably, members of the LARP6b and LARP6c subgroups harbored a PAM2 domain in the N-terminal region for which the consensus is “xxLxxxAxx(F/W)xP”. PAM2 appears to be coupled with the La-module to participate in RNA interactions [26, 54, 55].

The PABP protein binds to the poly(A) tail of mRNA and regulates mRNA stability [56]. Previous studies have demonstrated that the PAM2 motifs of AtLARP6b and AtLARP6c are required to mediate direct interaction with the MLLE domain of PABP [26]. In *Arabidopsis*, AtLARP6c functions as a RBP that is specific to pollen grains and pollen tubes, and guides the growth of pollen tubes towards the ovule by targeting transcripts with a B-box in the 5′-UTR. Binding of AtLARP6c, in a complex with the PABP, has been proposed to regulate trafficking and storage of its target transcripts [35]. In maize, ZmLARP6b and ZmLARP6c harbored a PAM2 domain at the N-terminus. Among these two proteins, the PAM2 of ZmLARP6c1 substitutes an F for an L residue at the N-terminal end, a substitution conserved in AtLARP6c (Fig. S4) [26]. However, the putative RNA-binding activity of ZmLARP6c1 in mature pollen and growing pollen tubes remains untested. In the present study, GO term enrichment analysis revealed that 204 (131 up- and 73 downregulated) and 61 (45 up- and 16 downregulated) annotated DEGs (FDR < 0.01) responded in the subset of genes involved in RNA binding in *Zmlarp6c1::Ds* and *ZmLARP6c1-*OE, respectively, compared with the WT (Table S6). As indicated above in the KEGG pathway analysis, the expression levels of *PABP1* and *PABP2* homologous genes were up- and downregulated in *Zmlarp6c1::Ds* and *ZmLARP6c1-*OE, respectively (Table S8), suggesting that expression of these genes is directly responsive to the level of *ZmLARP6c1*. One ortholog of *PABP1* (Zm00001eb095540, Zm00001eb426070) in *Arabidopsis* (*PAB4*; AT2G23350) is expressed in vegetative and reproductive tissues and functionally interacts with eIF4G and eIFiso4G2, and is required for growth and normal fertility [57]. An additional ortholog of *PABP1* (Zm00001eb386170), the *Arabidopsis* gene *PAB3* (AT1G22760), shows male gametophyte-specific expression [58] and co-localizes with the potential RNA-binding proteins ALBA4 and ALBA6 in the cytoplasm of mature pollen grains, possibly influencing mRNA metabolism [59]. Finally, an ortholog of *PABP2* (Zm00001eb034750, Zm00001eb079940) in *Arabidopsis*, AT3G12640, is consistently expressed in mature pollen grains, hydrated pollen grains, and pollen tubes, raising the possibility that it plays an important role in pollen germination [60]. We speculate that *Ds* insertion mutant or overexpression of *ZmLARP6c1* may affect pollen grain germination or pollen tube elongation by modulating expression of pollen-specific RNA-binding genes in maize. In the *Zmlarp6c1::Ds* mutant, the *tdsgR82C05* insertion is located in the fifth exon in the RRM-L3a domain coding region, which likely interferes with *ZmLARP6c1* function. The *Zmlarp6c1::Ds* mutant shows defective pollen transmission, which is associated with altered pollen germination dynamics and pollen tube elongation rates [36]. Hence, we suggest the La-module of ZmLARP6c1 is crucial for its role in pollen germination and pollen tube elongation, and hypothesize that ZmLARP6c1 RNA-binding activity is mediated by the composition of the La-module but is not dependent on PAM2. Further study is needed to clarify how the binding of ZmLARP6c1 with PABP contributes mechanistically to the regulation of its target transcripts that affect pollen germination and pollen tube growth.

Transcriptome analysis revealed that no other *ZmLARP* gene expressed in the pollen was up- or downregulated in *Zmlarp6c1::Ds* and *ZmLARP6c1-*OE relative to the WT. The qRT-PCR results of expression of *ZmLARP6s* genes in mutant or overexpression pollen and the corresponding WT (Fig. S10) were consistent with the RNA-seq results (Table S9). The ZmLARP6c2 protein, which is closely related to ZmLARP6c1, was weakly expressed in the pollen and the silk (Figs. 1 and 2). These results implied that the plants were unable to compensate for the mutation to *ZmLARP6c1* and that the two *ZmLARP6* genes may not function redundantly.

Methyl jasmonate- and ABA-responsive elements were the most abundant *cis*-acting elements associated with plant hormones in the promoter of *ZmLARP6c1* (Fig. S5). Under abiotic stress, the JA content in the plant increases and jasmonate isoleucine (JA-Ile) is formed by catalyzation of JAR1. The JA-Ile conjugate facilitates the interaction of JAZ with the F-box protein COI1 within the SKP1/CUL/F-box (SCF) complex, leading to the proteasomal degradation of JAZ. The SCF^COI1^-JA-Ile complex is formed to ubiquitinate JAZ proteins and release inhibition by the transcription factor MYC2, resulting in the expression of JA-responsive genes [61, 62]. In addition to its involvement in the response to abiotic stress, JA plays important and diverse roles in the plant life cycle, including plant growth and development, pollen germination, and flower formation [63–66]. The present GO and KEGG pathway analyses suggested that the DEGs were mainly enriched in terms involved in the JA biosynthetic process, JA metabolic process, JA-mediated signaling pathway, and response to JA (Fig. 7; Table S8). In comparison with the WT, in the JA metabolic process, *JAR1* (Zm00001eb101010) and *methylesterase* (Zm00001eb151170) homologous genes were upregulated in *ZmLARP6c1-*OE. The conversion between JA and JA-Ile is catalyzed by *JAR1*, and JA-Ile can be metabolized into MeJA dependent on *methylesterase*. In *Arabidopsis*, overexpression of *JAR1* increases the JA-Ile content and alters the hormonal profile to improve drought stress tolerance [67]. In *Camellia oleifera*, exogenous JA and MeJA inhibit pollen germination and pollen tube growth, and exogenous JA induces the expression of *COI1*, *JAZ1*, and *MYC2* in pollen [65]. We speculate that the induced expression of *JAR1* and *methylesterase* homologous genes in *ZmLARP6c1-*OE could change the contents of JA and its derivatives, as well as the expression of JA signaling pathway genes, and thus influence pollen germination in maize (Fig. 5). In the JA-mediated signaling pathway, two *COI1* homologous genes, Zm00001eb011780 (Zm00001d028543, *ZmCOI2a*) and Zm00001eb397990 (Zm00001d047848, *ZmCOI2b*), were up- and downregulated, respectively, in *Zmlarp6c1::Ds*. A previous report revealed that the maize *coi2a*, *coi2b*, and *coi2a coi2b* mutants exhibit severely defective pollen germination and altered expression of *ZmJAZ* genes [68]. In the present study, the functionally redundant genes *ZmCOI2a* and *ZmCOI2b* were up- and downregulated, respectively, in *Zmlarp6c1::Ds* pollen. However, the degree of *ZmCOI2b* downregulation (− 0.78) was stronger than the upregulation of *ZmCOI2a* (0.62). We speculated that knockdown of *ZmLARP6c1* decreased the total expression of *ZmCOI2* genes, thus inhibiting pollen germination and pollen tube growth in *Zmlarp6c1::Ds*. In addition, *JAZ* homologous genes were all downregulated in both *Zmlarp6c1::Ds* and *ZmLARP6c1-*OE, and *MYC2* homologous genes were up- and downregulated in both *Zmlarp6c1::Ds* and *ZmLARP6c1*-OE. The *JAZ* and *MYC* genes in the JA signaling pathway are reported to be involved in pollen germination. For instance, pollen viability and percentage pollen germination were significantly reduced in transgenic *Arabidopsis* overexpressing *GhWRKY22*, as a result of downregulation of *JAZ* gene expression [69]; overexpression of a MYC5-SRDX repressor might be activated by JA signaling to greatly reduce pollen germination and pollen tube length [70]. Moreover, ABA signaling pathway genes are involved in pollen germination and growth. In recent research, knockdown of *ABF.D.2* inhibited pollen tube growth by decreasing the expression levels of *LRX.A2.1* and *LRX.A2.2* in pear [71]. In the current study, an *ABF* homologous gene (Zm00001eb289080) was down-regulated in *Zmlarp6c1::Ds* (− 0.97) and upregulated in *ZmLARP6c1-*OE (1.14) (Fig. 8; Table S8). These results were consistent with the expression of ABF affecting pollen tube growth in plants. Taken together, we interpret that *Ds* insertion mutant or overexpression of *ZmLARP6c1* may affect pollen germination or pollen tube growth by modulating JA and ABA biosynthetic, metabolic, signaling pathway, and responsive genes in maize. Further study is needed to clarify how changes in gene expression contribute mechanistically to pollen germination and pollen tube growth.

## Conclusions

In the current investigation, we performed a genome-wide investigation of the LARP proteins in plant species. The LARP proteins were classified into three families based on a phylogenetic analysis. The similar exon length, the conserved motifs, the diverse *cis*-acting elements in the promoter and the expression patterns in various tissues of *ZmLARP* family shed light on their evolutionary characteristics and potential functions of *ZmLARP* genes in maize. In addition, overexpression of *ZmLARP6c1*, which specifically expressed in pollen and localized in nucleus and cytoplasm, enhanced the pollen germination rate thus raised the possibility that increased in *ZmLARP6c1* increased pollen resilience. The critical regulatory function of *ZmLARP6c1* in maize pollen germination can be achieved by modulating the expression of *PABP* homologous genes and genes involved in jasmonic acid and abscisic acid biosynthesis, metabolism, signaling pathways and response in a *ZmLARP6c1::Ds* mutant and *ZmLARP6c1*-overexpression line compared with the corresponding wild type.

### Supplementary Information

**Additional file 1: Supplementary Table S1.** Primers used in this study. **Supplementary Table S2. **Amino acid sequences of LARP proteins from eight plant species. **Supplementary Table S3.** Information on LARP proteins from eight plant species. **Supplementary Table S4.** *Cis*-acting elements in the promoter of LARP proteins in maize. **Supplementary Table S5. **Differentially expressed genes (FDR < 0.01) in Gene Ontology (GO) subcategories (q value < 0.05). **Supplementary Table S6. **Differentially expressed genes (FDR < 0.01) in the subset of genes involved in RNA binding and reproduction. **Supplementary Table S7.** Log-fold change and significance of differentially expressed genes in the subset of genes involved in pollen development. **Supplementary Table S8.** Log-fold change and significance of jasmonic acid, abscisic acid, and mRNA surveillance-related homologous genes detected in the pollen of the *Zmlarp6c1::Ds* and* ZmLARP6c1*-OE genotypes. **Supplementary Table S9.** FPKM of *ZmLARP6s* in RNA-seq.

**Additional file 2: Supplementary Fig. S1.** Gene structure and motif analysis of LARPs in maize. (A) Gene structure of *ZmLARP* genes in maize. Blue boxes represent exons, black lines represent introns, and gray boxes represent untranslated regions. (B) Motif composition of ZmLARP proteins in maize. Colored boxes represent conserved motifs detected in this study. **Supplementary Fig. S2.** Alignment of amino acid sequences of ZmLARP1 proteins. Red shading, LAM. **Supplementary Fig. S3.** Alignment of amino acid sequences of ZmLa proteins. Red shading, LAM; blue shading, RRM1; green shading, RRM2. **Supplementary Fig. S4.** Alignment of amino acid sequences ZmLARP6 proteins. Red shading, LAM; purple shading, RRM-L3a; yellow shading, LSA; pink shading, PAM2. **Supplementary Fig. S5.** Identification of potential *cis*-acting elements in the ZmLARP promoters in maize. (A) Different types of *cis*-acting elements are marked by differently colored rectangles. One the left is a phylogenetic tree for ZmLARP proteins. (B) Heatmap of *cis*-acting elements in the promoter of ZmLARP proteins. **Supplementary Fig. S6.** *In vitro* pollen germination and pollen tube length. (A) Germinated, non-germinated, and ruptured pollen grains of *Zmlarp6c1::Ds* compared with the wild type (WT) at 15 and 30 min after plating on pollen growth medium (PGM). Data are the mean ± SE (*n* = 4). (B) Representative fields-of-view of germinated pollen grains at 30 min after plating on PGM. (C) Pollen tube length of *Zmlarp6c1::Ds* and the WT at 30 min after plating on PGM. Data are the mean ± SD. * *P* < 0.05, ** *P* < 0.01, *** *P* < 0.001. **Supplementary Fig. S7.** Pollen grain diameter *ZmLARP6c1*-OE and the wild type (WT). At least 150 pollen grains were measured per replicate. All data are the means of four biological replicates and error bars indicate the SD. **Supplementary Fig. S8.** Classification of of DEGs in pollen of *Zmlarp6c1::Ds* and *ZmLARP6c1*-OE compared with WT by using Gene Ontology (GO) functional annotation from Venn diagrams. **Supplementary Fig. S9.** Expression analysis of jasmonic acid and abscisic acid biosynthetic, metabolic, mediated signaling pathway, and responsive genes in maize pollen evaluated by qRT-PCR. Data are means ± SE (*n* = 3). **Supplementary Fig. S10.** Expression analysis of *ZmLARP6s* genes in maize pollen evaluated by qRT-PCR. Data are means ± SE (*n* = 3).

**Additional file 3: Supplementary Fig. S11. **Gel electrophoresis image detection of *ZmLARP6c1*-OE transgenic lines by PCR. bar, bialaphos resistance gene; 1–5, *ZmLARP6c1*-OE1–OE5 lines; PC, positive control; MK, marker. **Supplementary Fig. S12.** Western blot image of *ZmLARP6c1*-OE lines. **Supplementary Fig. S13. **SDS-PAGE gel stained with Coomassie Blue image of total protein from WT and *ZmLARP6c1*-OE lines.

## Data Availability

The datasets generated and analyzed during the current study are available in the National Center for Biotechnology Information repository: https://www.ncbi.nlm.nih.gov/bioproject/ PRJNA1064632, accession number- PRJNA1064632.

## References

[1] Dedow LK, Bailey-Serres J (2019). Searching for a match: structure, function and application of sequence-specific RNA-binding proteins. Plant Cell Physiol.

[2] Kramer MC, Anderson SJ, Gregory BD (2018). The nucleotides they are a-changin': function of RNA binding proteins in post-transcriptional messenger RNA editing and modification in Arabidopsis. Curr Opin Plant Biol.

[3] Cho H, Cho HS, Hwang I (2019). Emerging roles of RNA-binding proteins in plant development. Curr Opin Plant Biol.

[4] Prall W, Sharma B, Gregory BD (2019). Transcription is just the beginning of gene expression regulation: the functional significance of RNA-binding proteins to post-transcriptional processes in plants. Plant Cell Physiol.

[5] Wolin SL, Cedervall T (2002). The La protein. Annu Rev Biochem.

[6] Bousquet-Antonelli C, Deragon JM (2009). A comprehensive analysis of the La-motif protein superfamily. RNA.

[7] Bayfield MA, Yang R, Maraia RJ (2010). Conserved and divergent features of the structure and function of La and La-related proteins (LARPs). Biochim Biophys Acta.

[8] Deragon JM (2021). Distribution, organization an evolutionary history of La and LARPs in eukaryotes. RNA Biol.

[9] Koster T, Marondedze C, Meyer K, Staiger D (2017). RNA-binding proteins revisited - the emerging Arabidopsis mRNA interactome. Trends Plant Sci.

[10] Maraia RJ, Mattijssen S, Cruz-Gallardo I, Conte MR (2017). The La and related RNA-binding proteins (LARPs): structures, functions, and evolving perspectives. Wiley Interdiscip Rev RNA.

[11] Maraia RJ, Intine RV (2001). Recognition of nascent RNA by the human La antigen: Conserved and diverged features of structure and function. Mol Cell Biol.

[12] Wolin SL, Cedervall T (2002). The La protein. Annu Rev Biochem.

[13] Curry S, Conte MR (2006). A terminal affair: 39-end recognition by the human La protein. Trends Biochem Sci.

[14] Maraia RJ, Bayfield MA (2006). The La protein-RNA complex surfaces. Mol Cell.

[15] Fleurdepine S, Deragon JM, Devic M, Guilleminot J, Bousquet-Antonelli C (2007). A bona fide La protein is required for embryogenesis in Arabidopsis thaliana. Nucleic Acids Res.

[16] Cui Y, Rao S, Chang B, Wang X, Zhang K, Hou X (2015). AtLa1 protein initiates IRES-dependent translation of WUSCHEL mRNA and regulates the stem cell homeostasis of Arabidopsis in response to environmental hazards. Plant Cell Environ.

[17] Zhang B, Jia J, Yang M, Yan C, Han Y (2012). Overexpression of a LAM domain containing RNA-binding protein LARP1c induces precocious leaf senescence in Arabidopsis. Mol Cells.

[18] Merret R, Descombin J, Juan YT, Favory JJ, Carpentier MC, Chaparro C (2013). XRN4 and LARP1 are required for a heat-triggered mRNA decay pathway involved in plant acclimation and survival during thermal stress. Cell Rep.

[19] Scarpin MR, Leiboff S, Brunkard JO (2020). Parallel global profiling of plant TOR dynamics reveals a conserved role for LARP1 in translation. Elife.

[20] Krueger BJ, Jeronimo C, Roy BB, Bouchard A, Barrandon C, Byers SA (2008). LARP7 is a stable component of the 7SK snRNP while P-TEFb, HEXIM1 and hnRNP A1 are reversibly associated. Nucleic Acids Res.

[21] Eichhorn CD, Chug R, Feigon J (2016). hLARP7 C-terminal domain contains an xRRM that binds the 3' hairpin of 7SK RNA. Nucleic Acids Res.

[22] Eichhorn CD, Yang Y, Repeta L, Feigon J (2018). Structural basis for recognition of human 7SK long noncoding RNA by the La-related protein Larp7. Proc Natl Acad Sci USA.

[23] Mennie AK, Moser BA, Nakamura TM (2018). LARP7-like protein Pof8 regulates telomerase assembly and poly(A)+TERRA expression in fission yeast. Nat Commun.

[24] Cruz-Gallardo I, Martino L, Kelly G, Atkinson RA, Trotta R, De Tito S (2019). LARP4A recognizes polyA RNA via a novel binding mechanism mediated by disordered regions and involving the PAM2w motif, revealing interplay between PABP, LARP4A and mRNA. Nucleic Acids Res.

[25] Aoki K, Adachi S, Homoto M, Kusano H, Koike K, Natsume T (2013). LARP1 specifically recognizes the 3' terminus of poly(A) mRNA. FEBS Lett.

[26] Merret R, Martino L, Bousquet-Antonelli C, Fneich S, Descombin J, Billey E (2013). The association of a La module with the PABP-interacting motif PAM2 is a recurrent evolutionary process that led to the neofunctionalization of La-related proteins. RNA.

[27] Lahr RM, Mack SM, Héroux A, Blagden S, Bousquet-Antonelli C, Deragon JM (2015). The La-related protein 1-specific domain repurposes HEAT-like repeats to directly bind a 5'TOP sequence. Nucleic Acids Res.

[28] Weng H, Kim C, Valavanis C, Wang Z, Schwartz LM (2009). Acheron, an novel LA antigen family member, binds to CASK and forms a complex with Id transcription factors. Cell Mol Biol Lett.

[29] Cai L, Fritz D, Stefanovic L, Stefanovic B (2010). Binding of LARP6 to the conserved 5’ stem-loop regulates translation of mRNAs encoding type I collagen. J Mol Biol.

[30] Vukmirovic M, Manojlovic Z, Stefanovic B (2013). Serine-threonine kinase receptor-associated protein (STRAP) regulates translation of type I collagen mRNAs. Mol Cell Biol.

[31] Manojlovic Z, Earwood R, Kato A, Perez D, Cabrera OA, Didier R (2017). A-related protein 6 controls ciliated cell differentiation. Cilia.

[32] Zhang Y, Stefanovic B (2016). LARP6 meets collagen mRNA: specific regulation of type I collagen expression. Int J Mol Sci.

[33] Cai L, Fritz D, Stefanovic L, Stefanovic B (2010). Nonmuscle myosin-dependent synthesis of type I collagen. J Mol Biol.

[34] Martino L, Pennell S, Kelly G, Busi B, Brown P, Atkinson RA, Salisbury (2015). Synergic interplay of the La motif, RRM1 and the interdomain linker of LARP6 in the recognition of collagen mRNA expands the RNA binding repertoire of the La module. Nucleic Acids Res.

[35] Billey E, Hafidh S, Cruz-Gallardo I, Litholdo CG, Jean V, Carpentier MC (2021). LARP6C orchestrates posttranscriptional reprogramming of gene expression during hydration to promote pollen tube guidance. Plant Cell.

[36] Zhou L, Vejlupkova Z, Warman C, Fowler JE (2021). A maize male gametophyte-specific gene encodes ZmLARP6c1, a potential RNA-binding protein required for competitive pollen tube growth. Front Plant Sci.

[37] Cheung AY, Wang H, Wu HM (1995). A floral transmitting tissue-specific glycoprotein attracts pollen tubes and stimulates their growth. Cell.

[38] Hulskamp M, Schneitz K, Pruitt RE (1995). Genetic evidence for a long-range activity that directs pollen tube guidance in Arabidopsis. Plant Cell.

[39] Johnson MA, Harper JF, Palanivelu R (2019). A fruitful journey: pollen tube navigation from germination to fertilization. Annu Rev Plant Biol.

[40] Honys D, Twell D (2004). Transcriptome analysis of haploid male gametophyte development in Arabidopsis. Genome Biol.

[41] Pina C, Pinto F, Feijo JA, Becker JD (2005). Gene family analysis of the Arabidopsis pollen transcriptome reveals biological implications for cell growth, division control, and gene expression regulation. Plant Physiol.

[42] Chettoor AM, Givan SA, Cole RA, Coker CT, Unger-Wallace E, Vejlupkova Z (2014). Discovery of novel transcripts and gametophytic functions via RNA-seq analysis of maize gametophytic transcriptomes. Genome Biol.

[43] Rutley N, Twell D (2015). A decade of pollen transcriptomics. Plant Reprod.

[44] Warman C, Panda K, Vejlupkova Z, Hokin S, Unger-Wallace E, Cole RA (2020). High expression in maize pollen correlates with genetic contributions to pollen fitness as well as with coordinated transcription from neighboring transposable elements. PLoS Genet.

[45] Fedoroff NV (2002). RNA-binding proteins in plants: the tip of an iceberg?. Curr Opin Plant Biol.

[46] Dreyfuss G, Kim VN, Kataoka N (2002). Messenger-RNA-binding proteins and the messages they carry. Nat Rev Mol Cell Biol.

[47] Chen C, Chen H, Zhang Y, Thomas HR, Frank MH, He Y, Xia R (2020). TBtools: an integrative toolkit developed for interactive analyses of big biological data. Mol Plant.

[48] Walley JW, Sartor RC, Shen Z, Schmitz RJ, Wu KJ, Urich MA (2016). Integration of omic networks in a developmental atlas of maize. Science.

[49] Jain N, Khurana P, Khurana JP (2023). AtTLP2, a Tubby-like protein, plays intricate roles in abiotic stress signalling. Plant Cell Rep.

[50] Bao Y, Song WM, Jin YL, Jiang CM, Yang Y, Li B (2014). Characterization of Arabidopsis Tubby-like proteins and redundant function of AtTLP3 and AtTLP9 in plant response to ABA and osmotic stress. Plant Mol Biol.

[51] Sato R, Maeshima M (2019). The ER-localized aquaporin SIP2;1 is involved in pollen germination and pollen tube elongation in Arabidopsis thaliana. Plant Mol Biol.

[52] Dock-Bregeon AC, Lewis KA, Conte MR (2021). The La-related proteins: structures and interactions of a versatile superfamily of RNA-binding proteins. RNA Biol.

[53] Thandapani P, O’Connor TR, Bailey TL, Richard S (2013). Defining the RGG/RG motif. Mol Cell.

[54] Alfano C, Sanfelice D, Babon J, Kelly G, Jacks A, Curry S (2004). Structural analysis of cooperative RNA binding by the La motif ans central RRM of human La protein. Nat Struct Mol Biol.

[55] Kotik-Kogan O, Valentine ER, Sanfelice D, Conte MR, Curry S (2008). Structural analysis reveals conformational plasticity in th erecognition of RNA 30 ends by the human La protein. Structure.

[56] Xie J, Kozlov G, Gehring K (2014). The ‘tale’ of poly(A) binding protein: the MLLE domain and PAM2-containing proteins. Biochim Biophys Acta Gene Regul Mech.

[57] Gallie DR (2018). Plant growth and fertility requires functional interactions between specific PABP and eIF4G gene family members. PLoS ONE.

[58] Belostotsky DA, Meagher RB (1993). Differential organ-specific expression of three poly(A)-binding protein genes from Arabidopsis thaliana. Proc Natl Acad Sci USA.

[59] Náprstková A, Kateřina Malínská K, Drábková LZ, Billey E, Náprstková D, Sýkorová E (2021). Characterization of ALBA family expression and localization in Arabidopsis thaliana generative organs. Int J Mol Sci.

[60] Wang Y, Zhang WZ, Song LF, Zou JJ, Su Z, Wu WH (2008). Transcriptome analyses show changes in gene expression to accompany pollen germination and tube growth in Arabidopsis. Plant Physiol.

[61] Katsir L, Chung HS, Koo AJ, Howe GA (2008). Jasmonate signaling: A conserved mechanism of hormone sensing. Curr Opin Plant Biol.

[62] Thines B, Katsir L, Melotto M, Niu Y, Mandaokar A, Liu G (2007). JAZ repressor proteins are targets of the SCF^COI1^ complex during jasmonate signalling. Nature.

[63] Campos ML, Kang JH, Howe GA (2014). Jasmonate-triggered plant immunity. J Chem Ecol.

[64] Feng MJ, Xu H, Zhang H, Zhu Y (2015). Recent progress in jasmonates regulation of plant growth and development. Plant Physiol J.

[65] Liu Y, Zhou J, Lu M, Yang J, Tan X (2022). The core jasmonic acid-signalling module CoCOI1/CoJAZ1/CoMYC2 are involved in Jas mediated growth of the pollen tube in *Camellia oleifera*. Curr Issues Mol Biol.

[66] Pak H, Guo Y, Chen MX, Chen KM, Li YL, Hua SJ (2009). The effect of exogenous methyl jasmonate on the flowering time, floral organ morphology, and transcript levels of a group of genes implicated in the development of oilseed rape flowers (*Brassica napus* L.). Planta.

[67] Mahmud S, Ullah C, Kortz A, Bhattacharyya S, Yu P, Gershenzon J (2022). Constitutive expression of JASMONATE RESISTANT 1 induces molecular changes that prime the plants to better withstand drought. Plant Cell Environ.

[68] Qi X, Guo S, Wang D, Zhong Y, Chen M, Chen C (2022). ZmCOI2a and ZmCOI2b redundantly regulate anther dehiscence and gametophytic male fertility in maize. Plant J.

[69] Wang Y, Li Y, He SP, Gao Y, Wang NN, Lu R (2019). A cotton (Gossypium hirsutum) WRKY transcription factor (GhWRKY22) participates in regulating anther/pollen development. Plant Physiol Biochem.

[70] Figueroa P, Browse J (2015). Male sterility in Arabidopsis induced by overexpression of a MYC5-SRDX chimeric repressor. Plant J.

[71] Wu L, Liu X, Zhang MY, Qi KJ, Jiang XT, Yao JL (2023). Self S-RNase inhibits ABF-LRX signaling to arrest pollen tube growth to achieve self-incompatibility in pear. Plant J.

