# Supplementary material for: Genome-wide investigation of the LARP gene family: focus on functional identification and transcriptome profiling of ZmLARP6c1 in maize pollen
Source: BMC Plant Biol. 2024 Apr 29;24:348. doi: 10.1186/s12870-024-05054-z (PMC11057080; doi:10.1186/s12870-024-05054-z)
Supplement: Supplementary file 2 — Additional file 2: Supplementary Fig. S1. Gene structure and motif analysis of LARPs in maize. (A) Gene structure of ZmLARP genes in maize. Blue boxes represent exons, black lines represent introns, and gray boxes represent untranslated regions. (B) Motif composition of ZmLARP proteins in maize. Colored boxes represent conserved motifs detected in this study. Supplementary Fig. S2. Alignment of amino acid sequences of ZmLARP1 proteins. Red shading, LAM. Supplementary Fig. S3. Alignment of amino acid sequences of ZmLa proteins. Red shading, LAM; blue shading, RRM1; green shading, RRM2. Supplementary Fig. S4. Alignment of amino acid sequences ZmLARP6 proteins. Red shading, LAM; purple shading, RRM-L3a; yellow shading, LSA; pink shading, PAM2. Supplementary Fig. S5. Identification of potential cis-acting elements in the ZmLARP promoters in maize. (A) Different types of cis-acting elements are marked by differently colored rectangles. One the left is a phylogenetic tree for ZmLARP proteins. (B) Heatmap of cis-acting elements in the promoter of ZmLARP proteins. Supplementary Fig. S6. In vitro pollen germination and pollen tube length. (A) Germinated, non-germinated, and ruptured pollen grains of Zmlarp6c1::Ds compared with the wild type (WT) at 15 and 30 min after plating on pollen growth medium (PGM). Data are the mean ± SE (n = 4). (B) Representative fields-of-view of germinated pollen grains at 30 min after plating on PGM. (C) Pollen tube length of Zmlarp6c1::Ds and the WT at 30 min after plating on PGM. Data are the mean ± SD. * P < 0.05, ** P < 0.01, *** P < 0.001. Supplementary Fig. S7. Pollen grain diameter ZmLARP6c1-OE and the wild type (WT). At least 150 pollen grains were measured per replicate. All data are the means of four biological replicates and error bars indicate the SD. Supplementary Fig. S8. Classification of of DEGs in pollen of Zmlarp6c1::Ds and ZmLARP6c1-OE compared with WT by using Gene Ontology (GO) functional annotation from Venn diagrams. Suppl [file 12870_2024_5054_MOESM2_ESM.pptx]

## Slide 1
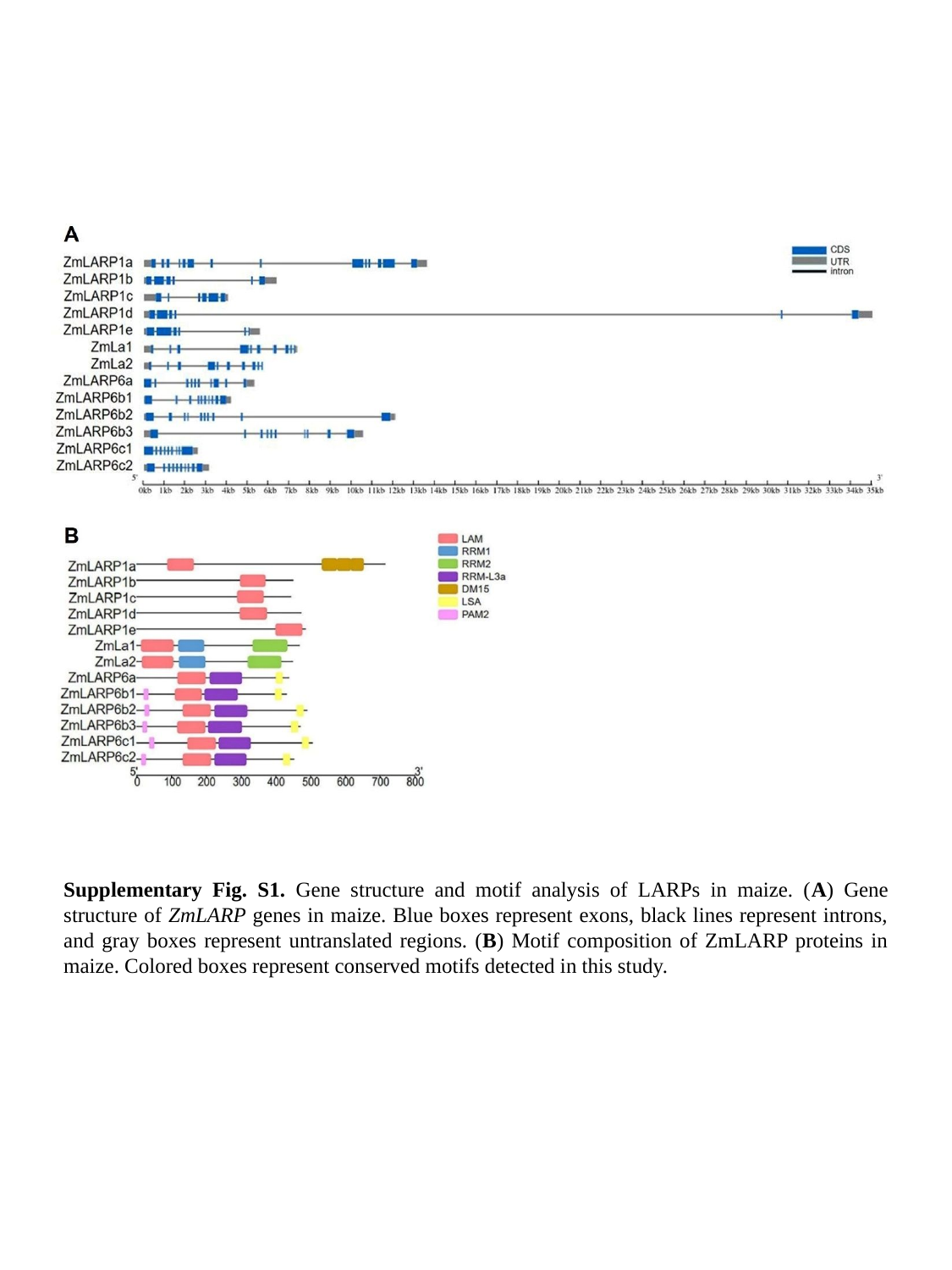

Supplementary Fig. S1. Gene structure and motif analysis of LARPs in maize. (A) Gene structure of ZmLARP genes in maize. Blue boxes represent exons, black lines represent introns, and gray boxes represent untranslated regions. (B) Motif composition of ZmLARP proteins in maize. Colored boxes represent conserved motifs detected in this study.

## Slide 2
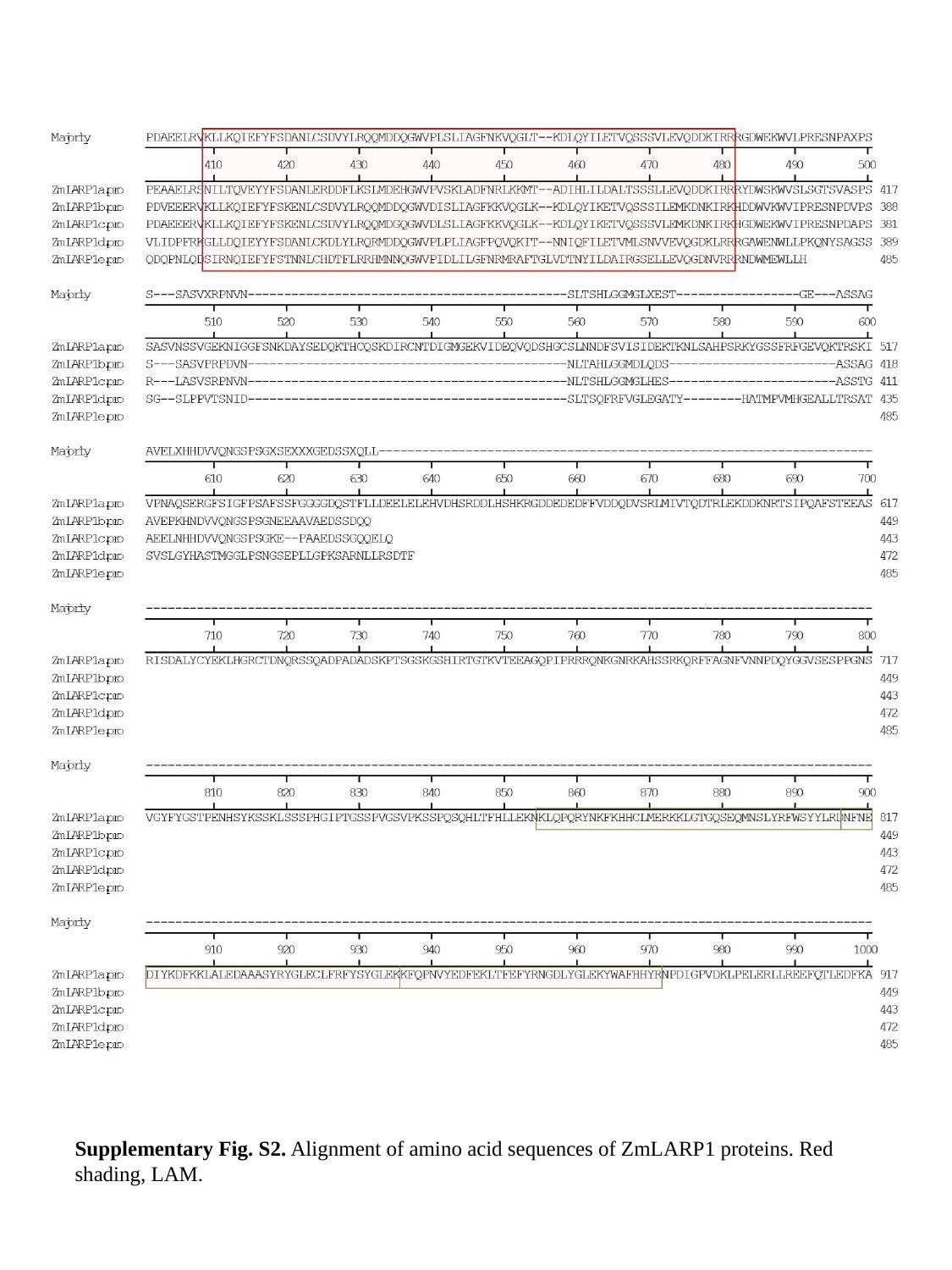

Supplementary Fig. S2. Alignment of amino acid sequences of ZmLARP1 proteins. Red shading, LAM.

## Slide 3
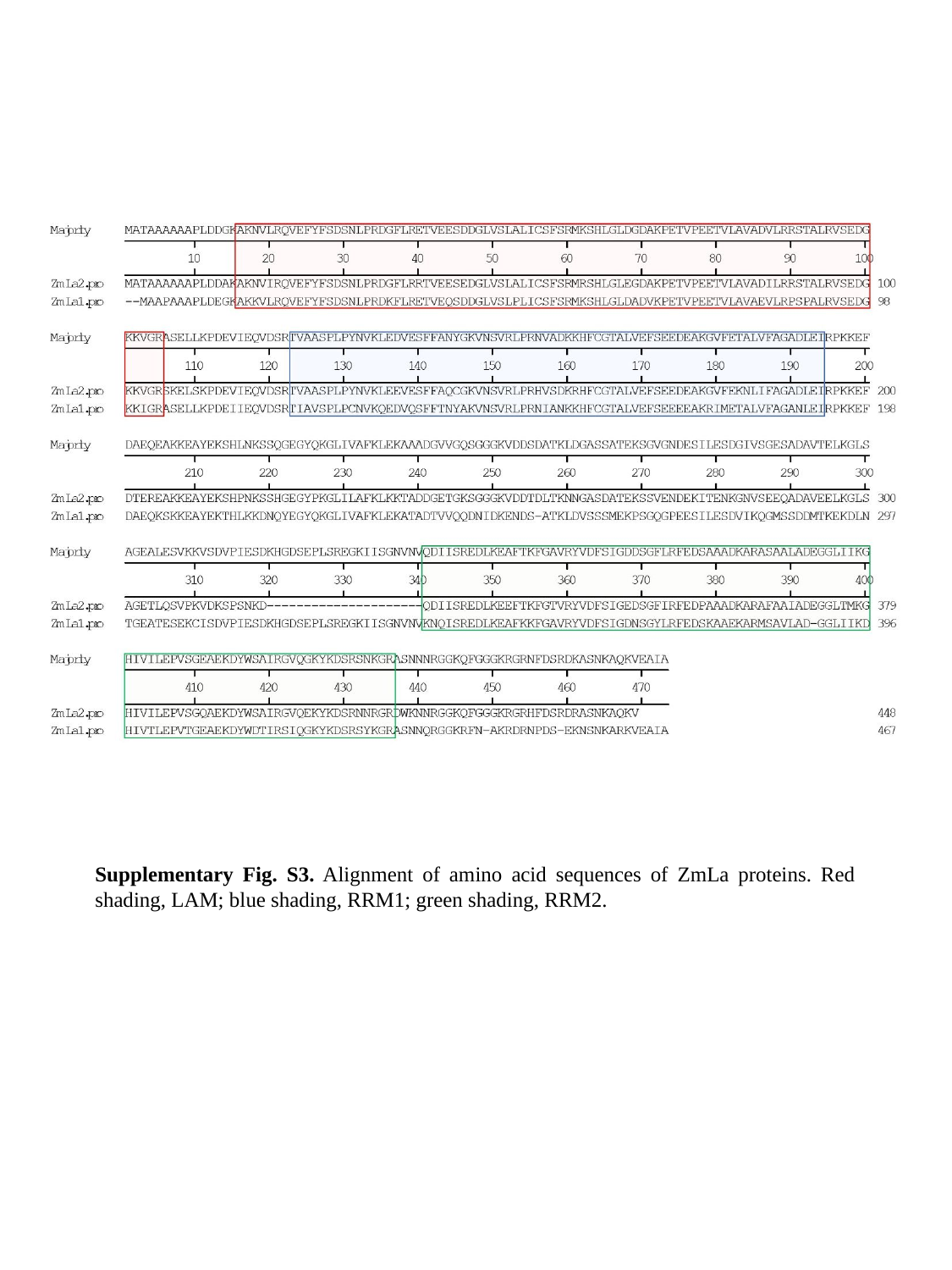

Supplementary Fig. S3. Alignment of amino acid sequences of ZmLa proteins. Red shading, LAM; blue shading, RRM1; green shading, RRM2.

## Slide 4
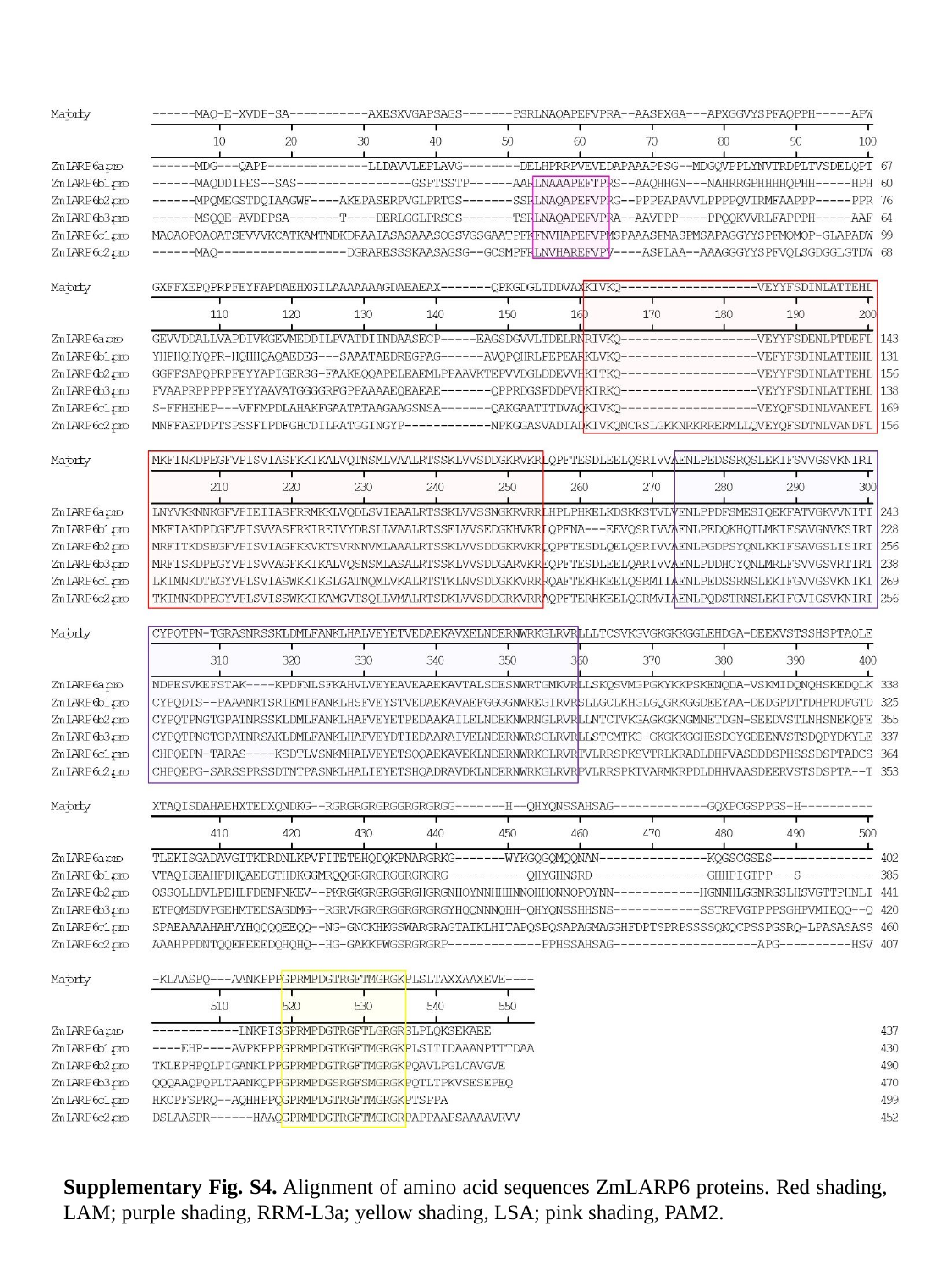

Supplementary Fig. S4. Alignment of amino acid sequences ZmLARP6 proteins. Red shading, LAM; purple shading, RRM-L3a; yellow shading, LSA; pink shading, PAM2.

## Slide 5
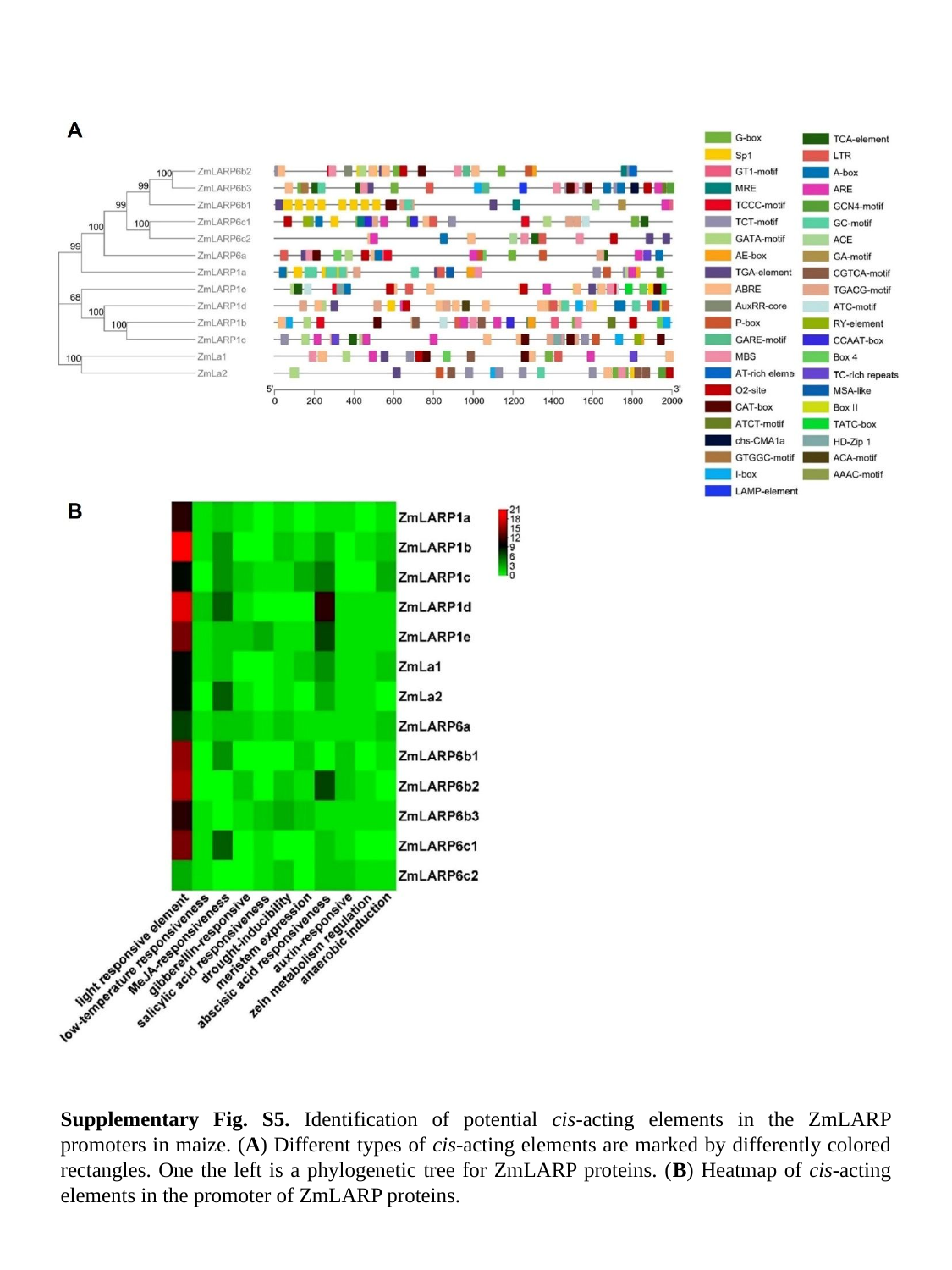

Supplementary Fig. S5. Identification of potential cis-acting elements in the ZmLARP promoters in maize. (A) Different types of cis-acting elements are marked by differently colored rectangles. One the left is a phylogenetic tree for ZmLARP proteins. (B) Heatmap of cis-acting elements in the promoter of ZmLARP proteins.

## Slide 6
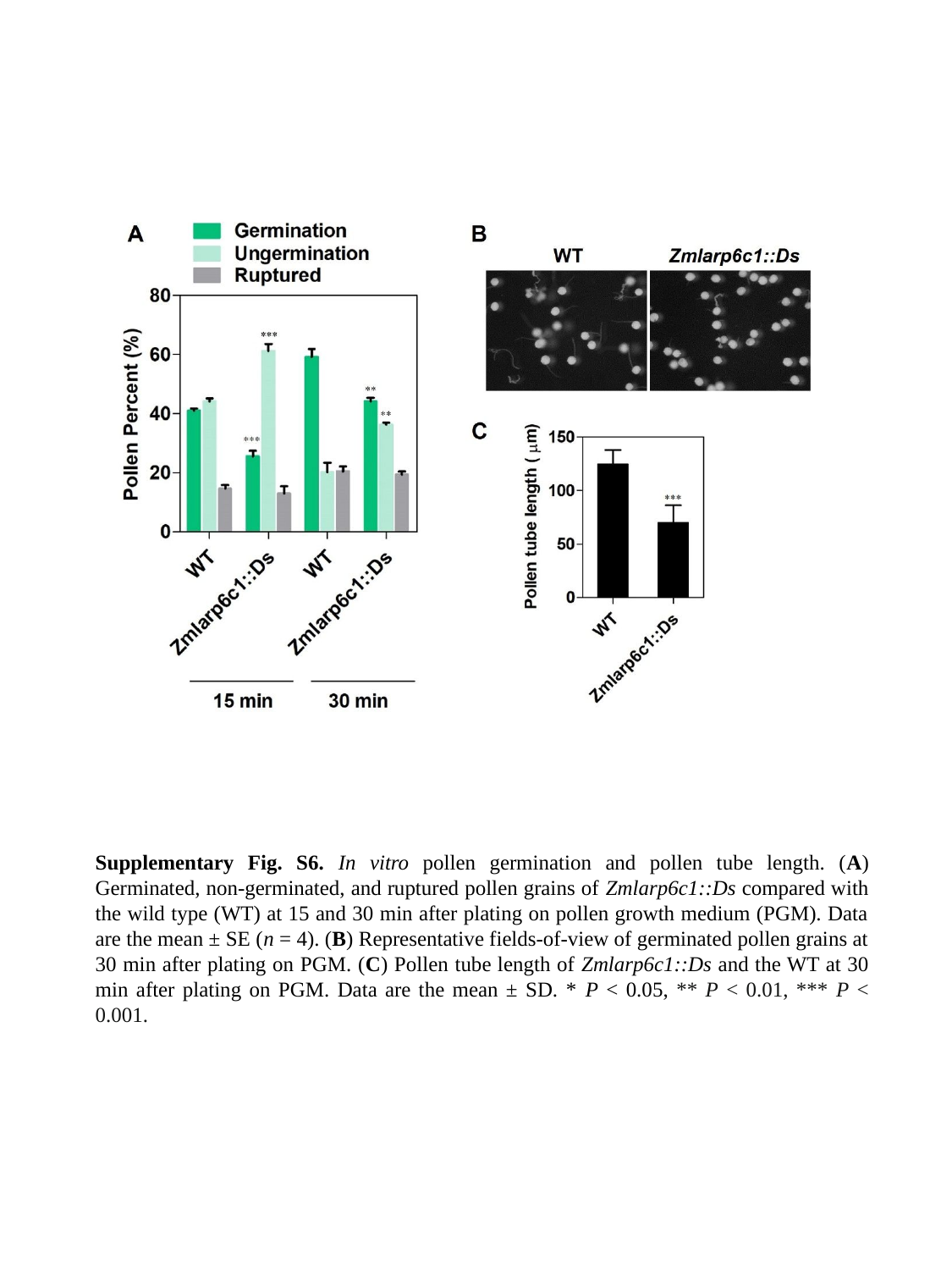

Supplementary Fig. S6. In vitro pollen germination and pollen tube length. (A) Germinated, non-germinated, and ruptured pollen grains of Zmlarp6c1::Ds compared with the wild type (WT) at 15 and 30 min after plating on pollen growth medium (PGM). Data are the mean ± SE (n = 4). (B) Representative fields-of-view of germinated pollen grains at 30 min after plating on PGM. (C) Pollen tube length of Zmlarp6c1::Ds and the WT at 30 min after plating on PGM. Data are the mean ± SD. * P < 0.05, ** P < 0.01, *** P < 0.001.

## Slide 7
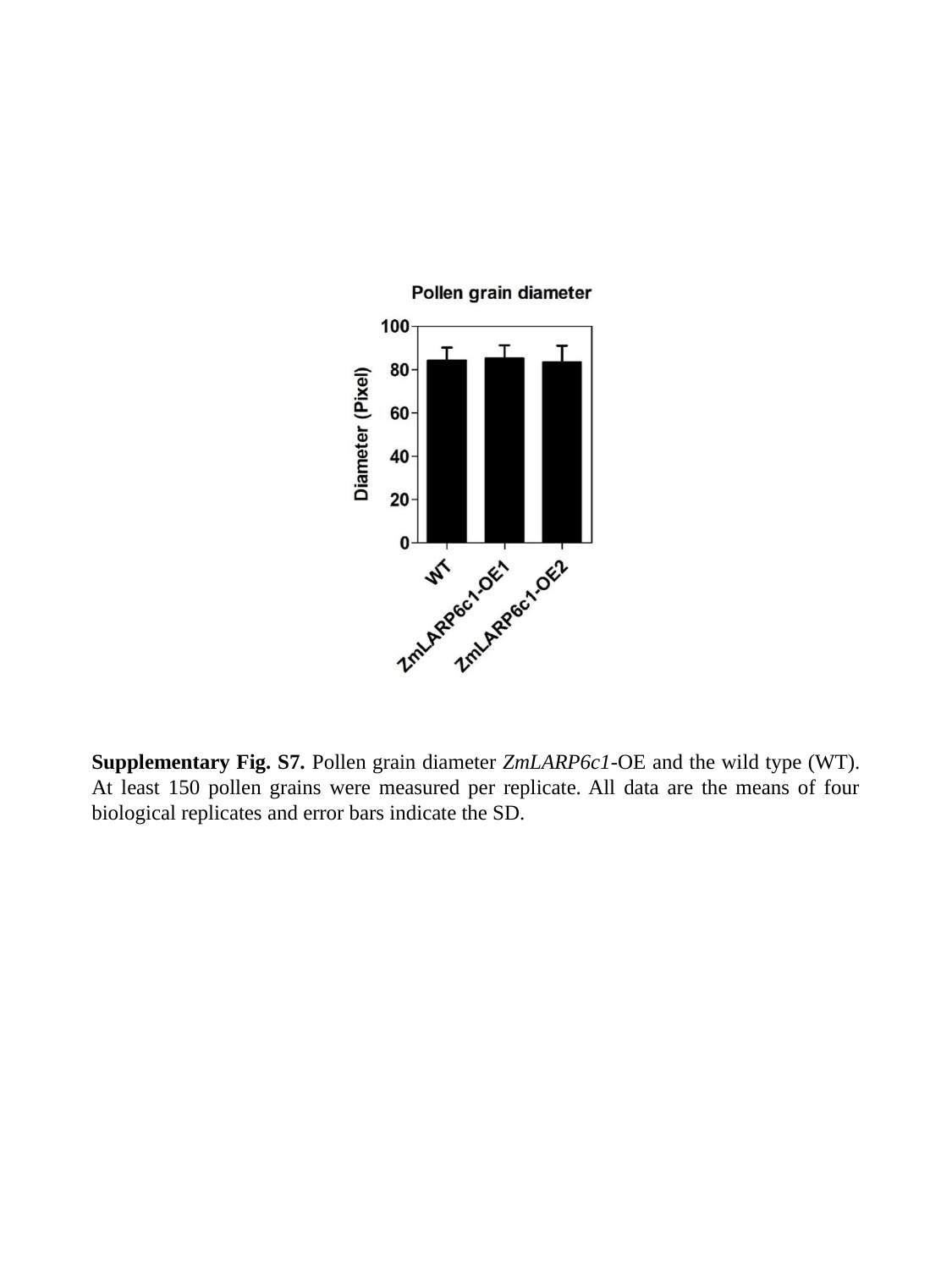

Supplementary Fig. S7. Pollen grain diameter ZmLARP6c1-OE and the wild type (WT). At least 150 pollen grains were measured per replicate. All data are the means of four biological replicates and error bars indicate the SD.

## Slide 8
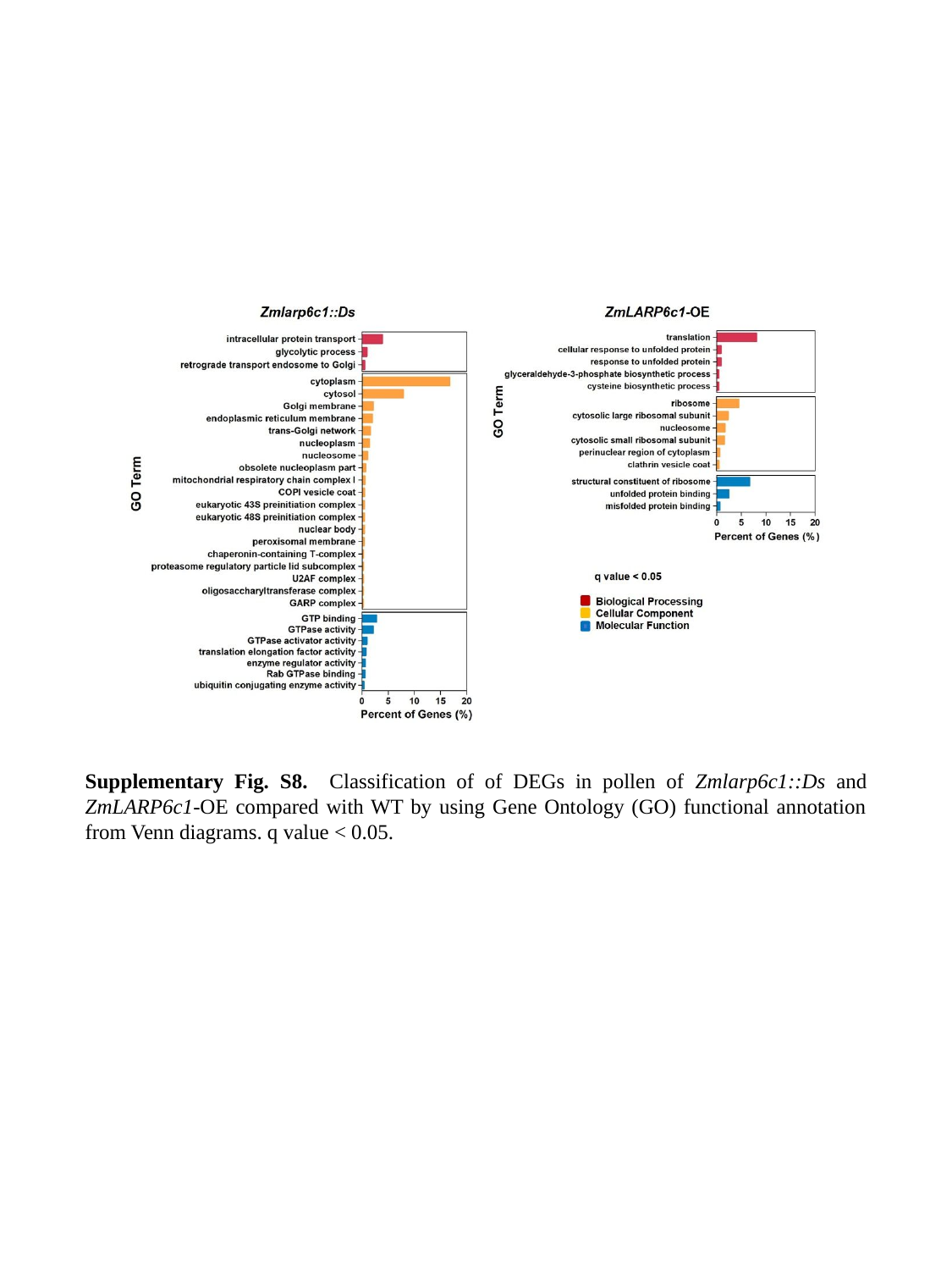

Supplementary Fig. S8. Classification of of DEGs in pollen of Zmlarp6c1::Ds and ZmLARP6c1-OE compared with WT by using Gene Ontology (GO) functional annotation from Venn diagrams. q value < 0.05.

## Slide 9
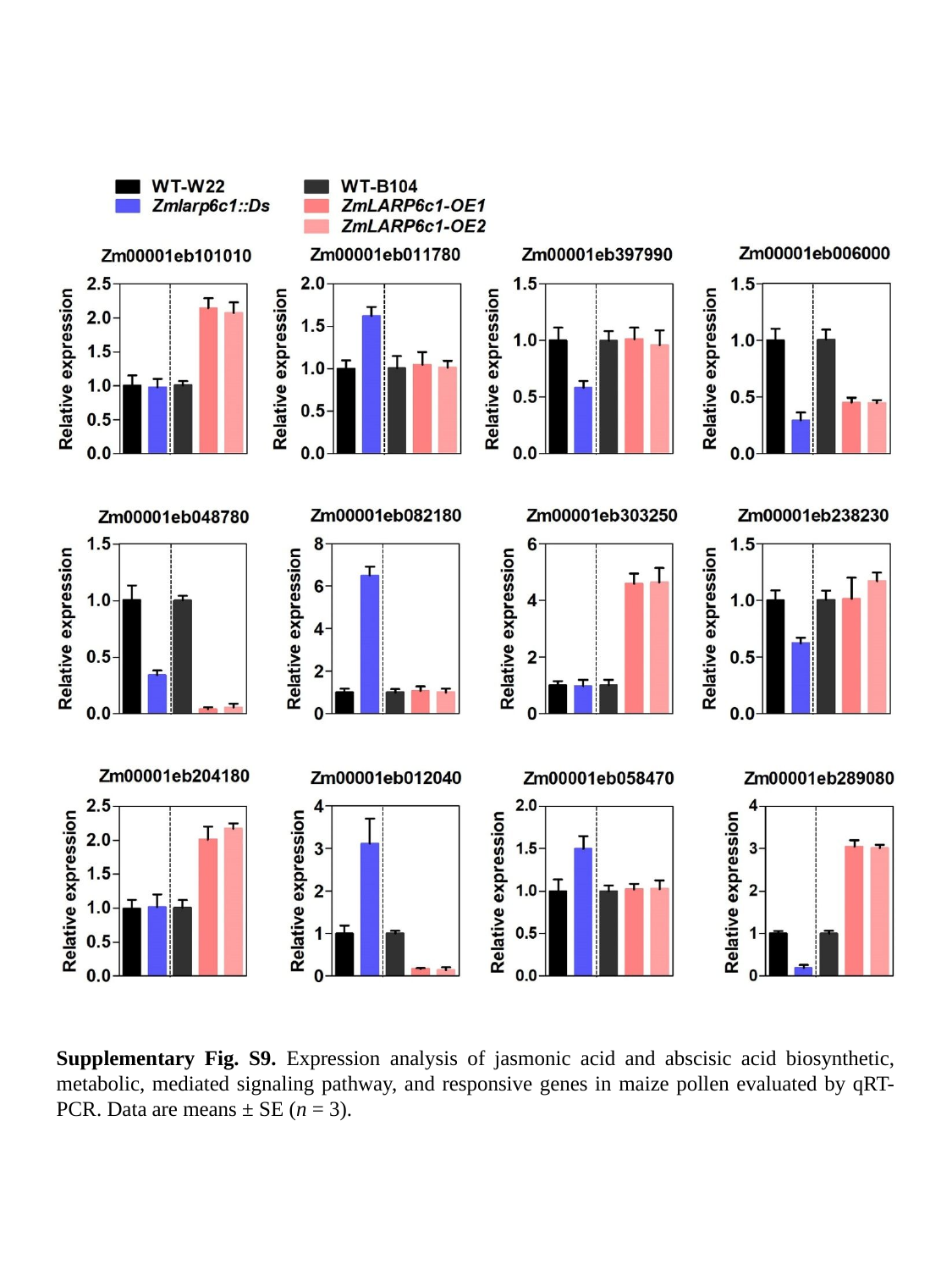

Supplementary Fig. S9. Expression analysis of jasmonic acid and abscisic acid biosynthetic, metabolic, mediated signaling pathway, and responsive genes in maize pollen evaluated by qRT-PCR. Data are means ± SE (n = 3).

## Slide 10
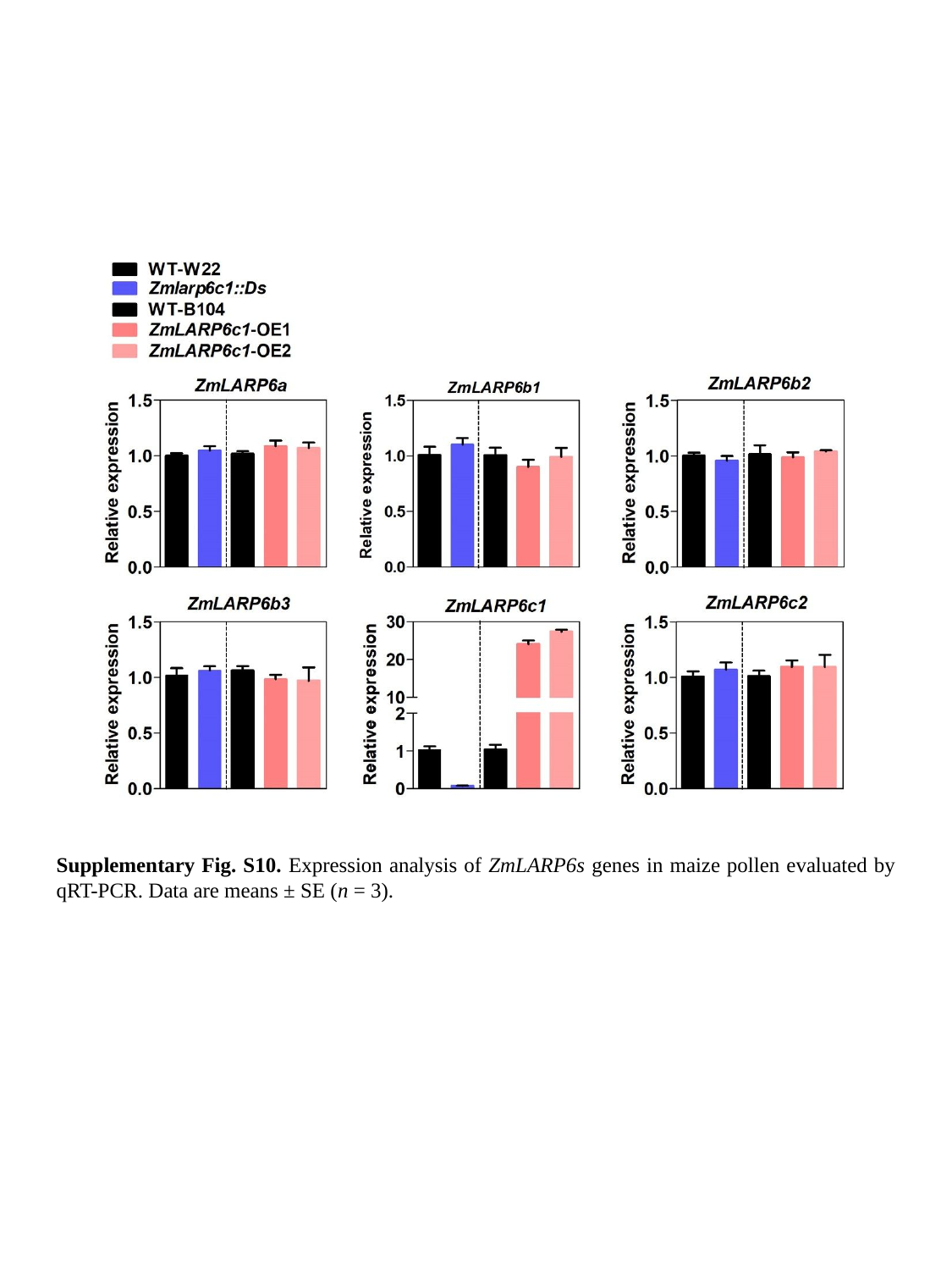

Supplementary Fig. S10. Expression analysis of ZmLARP6s genes in maize pollen evaluated by qRT-PCR. Data are means ± SE (n = 3).
