# Supplementary material for: Genome-wide investigation of the LARP gene family: focus on functional identification and transcriptome profiling of ZmLARP6c1 in maize pollen
Source: BMC Plant Biol. 2024 Apr 29;24:348. doi: 10.1186/s12870-024-05054-z (PMC11057080; doi:10.1186/s12870-024-05054-z)
Supplement: Supplementary file 3 — Additional file 3: Supplementary Fig. S11. Gel electrophoresis image detection of ZmLARP6c1-OE transgenic lines by PCR. bar, bialaphos resistance gene; 1–5, ZmLARP6c1-OE1–OE5 lines; PC, positive control; MK, marker. Supplementary Fig. S12. Western blot image of ZmLARP6c1-OE lines. Supplementary Fig. S13. SDS-PAGE gel stained with Coomassie Blue image of total protein from WT and ZmLARP6c1-OE lines. [file 12870_2024_5054_MOESM3_ESM.pptx]

## Slide 1
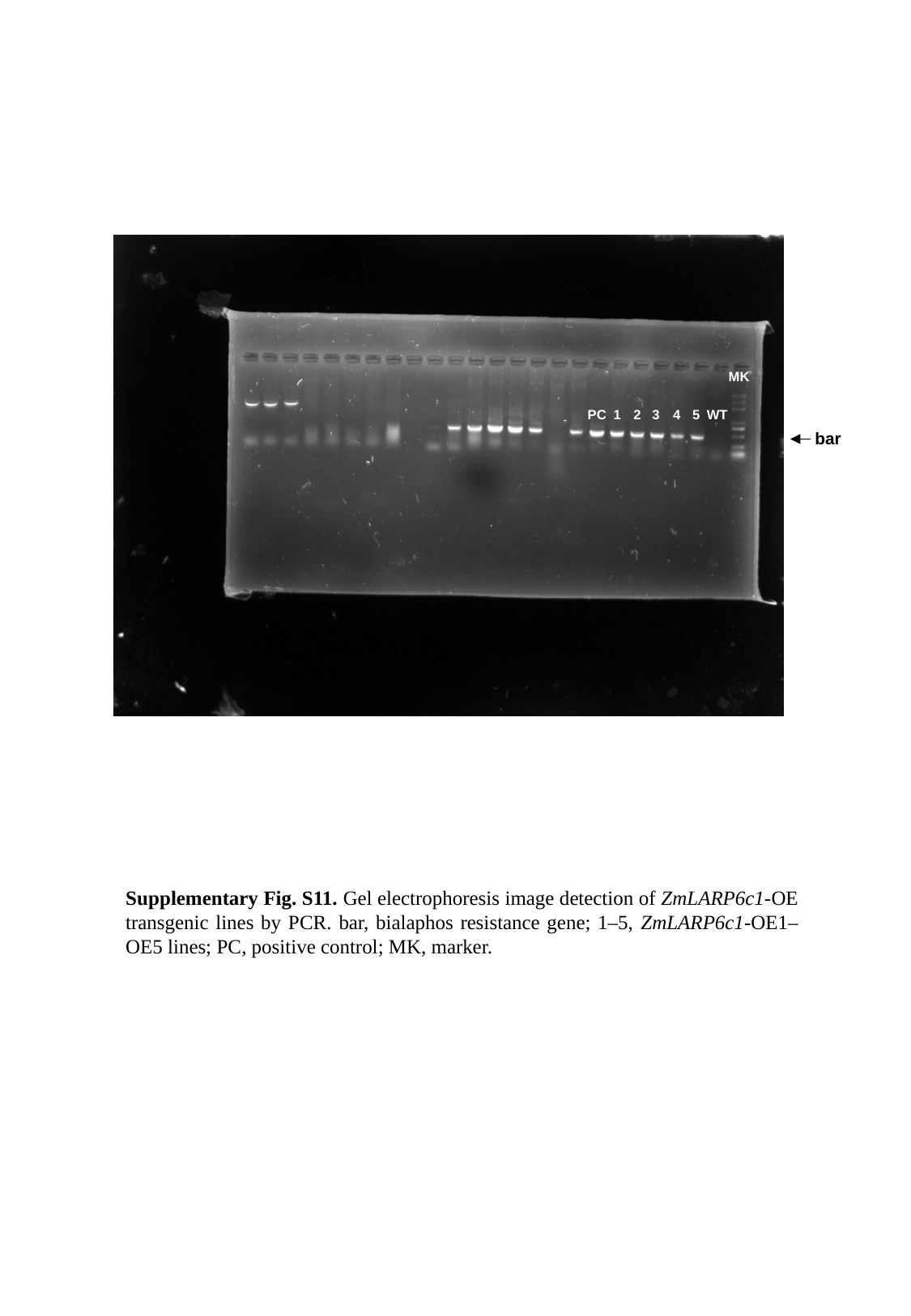

MK
PC
1
2
3
4
5
WT
bar
Supplementary Fig. S11. Gel electrophoresis image detection of ZmLARP6c1-OE transgenic lines by PCR. bar, bialaphos resistance gene; 1–5, ZmLARP6c1-OE1–OE5 lines; PC, positive control; MK, marker.

## Slide 2
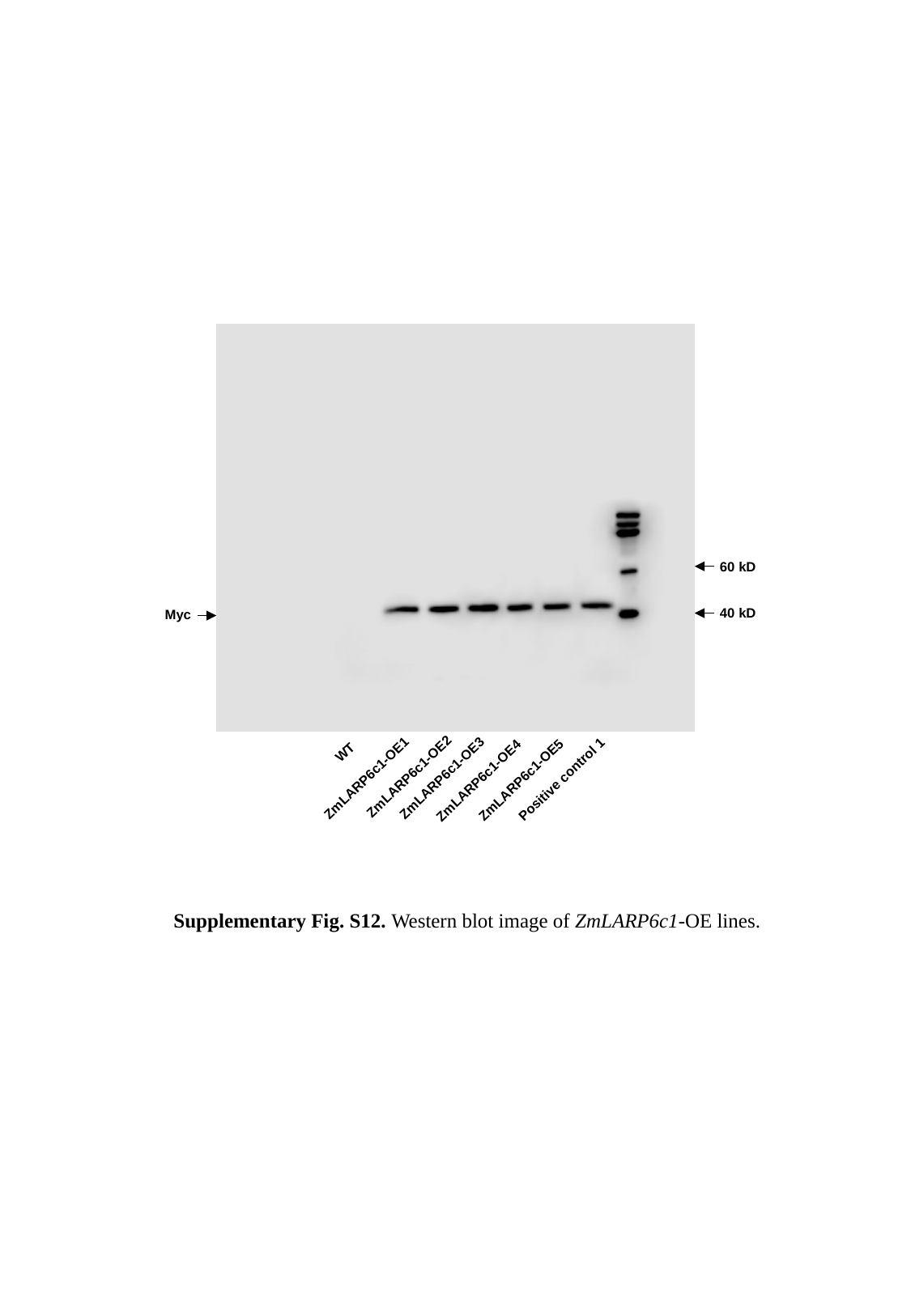

60 kD
40 kD
Myc
WT
ZmLARP6c1-OE2
ZmLARP6c1-OE3
ZmLARP6c1-OE1
ZmLARP6c1-OE5
ZmLARP6c1-OE4
Positive control 1
Supplementary Fig. S12. Western blot image of ZmLARP6c1-OE lines.

## Slide 3
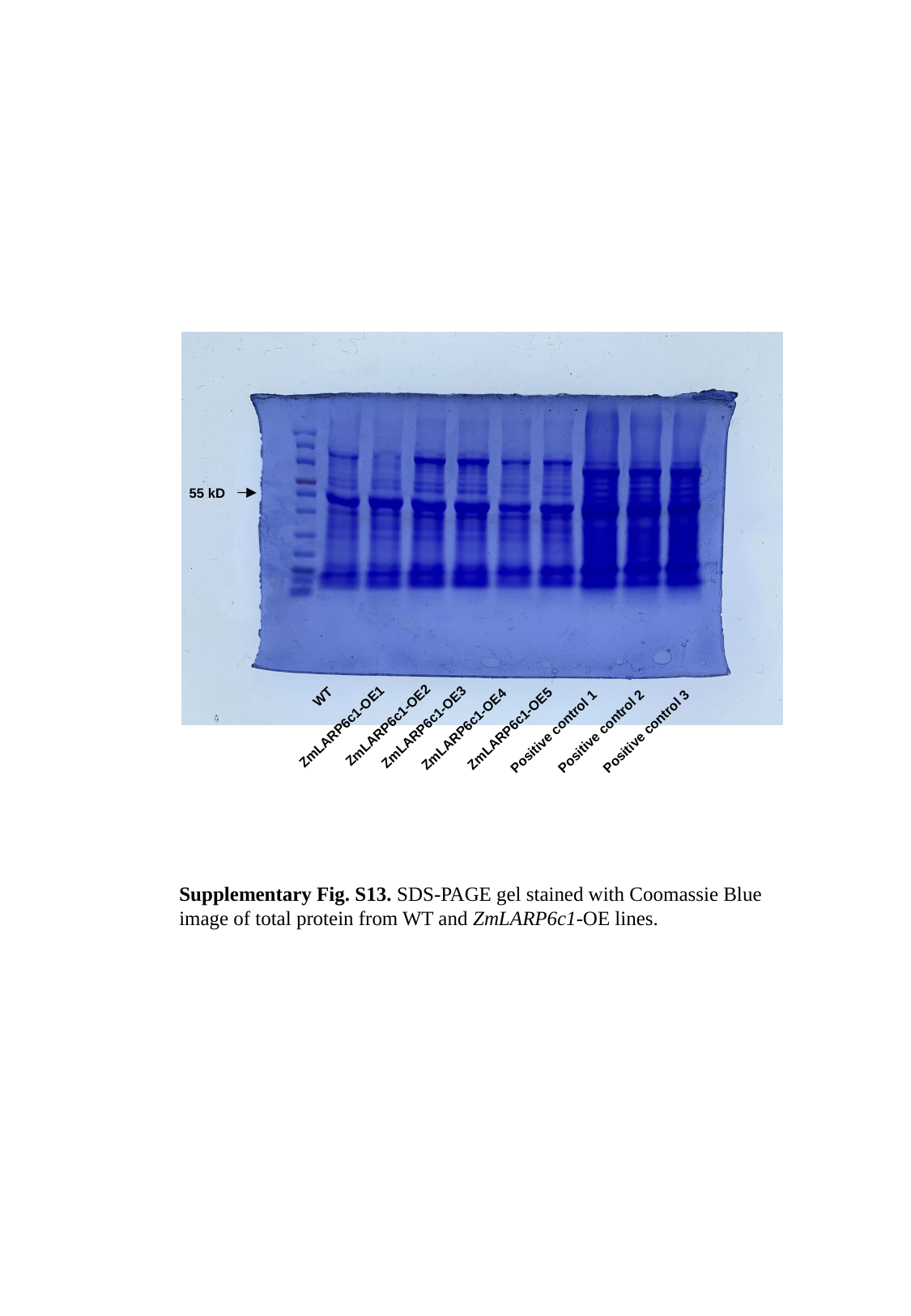

55 kD
WT
ZmLARP6c1-OE2
ZmLARP6c1-OE3
ZmLARP6c1-OE1
ZmLARP6c1-OE5
ZmLARP6c1-OE4
Positive control 1
Positive control 2
Positive control 3
Supplementary Fig. S13. SDS-PAGE gel stained with Coomassie Blue image of total protein from WT and ZmLARP6c1-OE lines.
